# The bHLH transcription factor gene *EGL3* accounts for the natural diversity in Arabidopsis fruit trichome pattern and morphology

**DOI:** 10.1093/plphys/kiae673

**Published:** 2024-12-22

**Authors:** Belén Méndez-Vigo, Noelia Arteaga, Alba Murillo-Sánchez, Sonia Alba, Carlos Alonso-Blanco

**Affiliations:** Departamento de Genética Molecular de Plantas, Centro Nacional de Biotecnología (CNB), Consejo Superior de Investigaciones Científicas (CSIC), Madrid 28049, Spain; Departamento de Genética Molecular de Plantas, Centro Nacional de Biotecnología (CNB), Consejo Superior de Investigaciones Científicas (CSIC), Madrid 28049, Spain; Departamento de Genética Molecular de Plantas, Centro Nacional de Biotecnología (CNB), Consejo Superior de Investigaciones Científicas (CSIC), Madrid 28049, Spain; Departamento de Genética Molecular de Plantas, Centro Nacional de Biotecnología (CNB), Consejo Superior de Investigaciones Científicas (CSIC), Madrid 28049, Spain; Departamento de Genética Molecular de Plantas, Centro Nacional de Biotecnología (CNB), Consejo Superior de Investigaciones Científicas (CSIC), Madrid 28049, Spain

## Abstract

The number and distribution of trichomes, i.e. the trichome pattern, in different plant organs show a conspicuous inter- and intraspecific diversity across Angiosperms that is presumably involved in adaptation to numerous environmental factors. The genetic and molecular mechanisms accounting for the evolution of trichome patterns have just begun to be elucidated. In this study, we aimed to identify and characterize *MALAMBRUNO 1* (*MAU1*), a locus affecting trichome number in the fruits of Arabidopsis (*Arabidopsis thaliana*) natural populations. To this end, we developed introgression lines (ILs) from the hairy fruit accession Doñana (Don-0) in the genetic background of the L*er* strain with glabrous fruits. Genetic analyses of ILs showed that *MAU1* affects fruit trichome patterns through synergistic epistasis with the MYB genes *TRICHOMELESS1* (*TCL1*), *GLABRA1* (*GL1*), and *TRIPTYCHON* (*TRY*). In addition, fine mapping and characterization of transgenic lines demonstrated that *MAU1* is the bHLH transcription factor gene *EGL3*, for which Don-0 carries a gain-of-function semidominant allele. Gene expression analyses did not detect differences between *EGL3* alleles, thus supporting that a structural missense mutation is the causal nucleotide polymorphism of Don-0. Further phylogenetic analyses of *EGL3* showed that most Arabidopsis populations with hairy fruits belong to 3 haplogroups, suggesting that additional *EGL3* natural alleles account for fruit trichome development. Finally, the characterization of *EGL3* pleiotropy indicates that Don-0 hyperfunction also increases stem trichome branching. We conclude that *EGL3* interactions in the core gene regulatory network of trichome development explain the Arabidopsis natural diversity for fruit trichome pattern and morphology.

## Teaser

*ENHANCER OF GLABRA3* interactions in the gene regulatory network of trichome development explain the diversity of trichome pattern and morphology in Arabidopsis fruits.

## Introduction

Trichome pattern, defined as the distribution and amount of trichomes (plant hairs) in different aerial organs, shows prominent diversity among and within plant species. In particular, the development of trichomes in specific organs, such as leaves, stems, or fruits, has been classically used as robust qualitative taxonomic traits that differentiate Angiosperm plants (Tutin et al. 1993; Judd et al. 1999). In addition, trichome pattern often displays qualitative and quantitative variation among natural populations of the same species (Hauser 2014). Much of this diversity is presumably involved in adaptation to multiple abiotic and biotic environmental factors, because trichomes have been shown to protect plants against drought and UV radiation, as well as from herbivore insects and pathogens (Fürstenberg-Hägg et al. 2013; Bickford 2016). However, despite its adaptive relevance, the genetic and molecular mechanisms underlying the natural diversity and evolution of trichome pattern are just beginning to be unraveled.

The molecular mechanisms of trichome development have been extensively studied in the model plant *Arabidopsis thaliana* (from here, Arabidopsis), which develops unicellular trichomes that are branched in leaves, simple and branched in the basal stem internodes, and only simple in sepals. By contrast, the upper stem internodes, pedicels, and fruits of Arabidopsis have been classically described as glabrous (Hülskamp and Schnittger 1998; Al-Shehbaz and O’Kane 2002), but it has been recently shown that this is not always the case (Arteaga et al. 2021, 2022). The study of artificially induced mutants in more than 140 genes has uncovered the main processes involved in the two trichome developmental phases. First, trichome patterning is defined by a core gene network that regulates the homeodomain (HD) transcription factor GLABRA2 (GL2), which triggers trichome fate in epidermal cells (Balkunde et al. 2010; Pattanaik et al. 2014; Wang and Chen 2014). *GL2* expression is directly activated by a trimeric complex encoded by 3 families of transcriptional regulators: the R2R3 MYB transcription factor GLABRA1 (GL1); the bHLH regulators GLABRA3 (GL3) and ENHANCER OF GLABRA3 (EGL3); and the WD40 protein TRANSPARENT TESTA GLABRA1 (TTG1). Moreover, single repeat R3 MYB transcription factors encoded by 7 genes repress trichome initiation by interfering with the function of the trimeric activation complex. Subsequently, trichome branching is controlled by multiple genes affecting cell expansion and endoreduplication (Balkunde et al. 2010; Fambrini and Pugliesi 2019).

Intricate interactions have been shown in the trichome regulatory network of Arabidopsis because several genes affect both phases, trichome patterning and branching (Folkers et al. 1997; Schellmann et al. 2002; Kirik et al. 2005; Pesch et al. 2014, 2015; Doroshkov et al. 2019), and some of them also display pleiotropy on various epidermal traits, including anthocyanin biosynthesis, seed coat flavonoid and mucilage production, or root hair differentiation (Zhang et al. 2003; Ishida et al. 2008; Lloyd et al. 2017; Zhang and Hülskamp 2019). Such pleiotropic effects have been explained by the combination of different R2R3 MYB and bHLH transcription factors to form distinct trimeric complexes that activate specific cellular processes in the epidermis (Gonzalez et al. 2008; Morohashi and Grotewold 2009). In this combinatorial model, at least eight R2R3 MYB transcription factors provide the trait specificity, but they interact with only three bHLH proteins (Zhang and Hülskamp 2019). Accordingly, mutations in MYB genes display less pleiotropy than those in *GL3* and *EGL3*, the later affecting trichome patterning and branching, as well as pigment biosynthesis and other epidermal traits (Zhang et al. 2003; Morohashi et al. 2007; Gonzalez et al. 2008).

Additional studies in other plants with different trichome morphology, such as the fibers of cotton, or the multicellular and glandular hairs of rice, cucumber, and tomato, are now addressing the functional conservation of genes and interactions of Arabidopsis regulatory network (Chalvin et al. 2020; Schuurink and Tissier 2020; Han et al. 2022; Wang et al. 2022). Overall, a substantial conserved function of the trimeric complex that activates trichome development has been found among phylogenetically distant species (Zhang and Hülskamp 2019). Moreover, comparative studies have shown that numerous genes encoding MYB, bHLH, C2H2 zinc finger, and HD transcription factors have analogous functions in the regulation of trichome patterning in different wild and crop plants (Zhang and Hülskamp 2019; Han et al. 2022). However, several regulatory mechanisms also appear as specific of particular plant lineages, as illustrated with the recent identification of another trimeric complex encoded by C2H2 and HD transcription factors in cucumber (Yang et al. 2022a). Therefore, a partial conservation of the trichome gene regulatory network has emerged across Angiosperms, although the extent of parallel and divergent trichome evolution is just beginning to be defined (Serna and Martin 2006; Chalvin et al. 2020; Han et al. 2022).

In contrast to the broad knowledge on the regulation of trichome development in several plants, only 10 different genes have been demonstrated to account for the natural diversity for trichome pattern in different organs and species. Most studies have addressed the qualitative intraspecific variation between completely glabrous and hairy individuals of wild and cultivated Brassicaceae plants, demonstrating that *GL1* accounts for the parallel evolution of this trait in numerous species with branched and unbranched trichomes (Hauser et al. 2001; Kivimäki et al. 2007; Li et al. 2013; Xuan et al. 2020; Meng et al. 2023; Fuster-Pons et al. 2024). Interestingly, another R2R3 MYB transcription factor also is responsible of the glabrous phenotype of nectarine varieties of *Prunus persica* (Yang et al. 2022b). In addition, the R3 MYB genes, *ENHANCER OF TRY AND CPC2* (*ETC2*), and *TRICHOMLESS1* (*TCL1*), as well as the bHLH gene *MYC1*, contribute to the quantitative variation for the amount of trichomes in leaves of Arabidopsis (Hilscher et al. 2009; Symonds et al. 2011; Arteaga et al. 2022). Three other genes, *DENSE PUBESCENCE1* (*Pd1*), *SPARSE PUBESCENCE* (*Ps*), and *GLABROUS PUBESCENCE* (*P1*) have been identified causing similar intraspecific variation in soybean leaves (Liu et al. 2020). However, the diversity for trichome pattern in organs other than leaves has just begun to be dissected in *Antirrhinum*, *Solanum*, and Arabidopsis. The analysis of several *Antirrhinum* and *Solanum* species has shown that inter- and intraspecific diversity for the development of trichomes in leaves and stem internodes is caused by *HAIRY* (*H*) and *HAIRS ABSENT* (*H*), respectively, which encode an epidermal-specific glutaredoxin and a C2H2 zinc finger transcription factor (Tan et al. 2020; Gasparini et al. 2023).

Furthermore, it has recently been found that multiple Arabidopsis populations also develop trichomes on fruits, pedicels, and upper stem internodes (Arteaga et al. 2021, 2022). This trichome pattern appears as an evolutionary innovation likely involved in adaptation to the low precipitation of southern Europe because it has exclusively evolved in the ancient relict lineage of the Iberian Peninsula, which diverged from the Eurasian nonrelict populations ∼90 to 60 kya (1001 Genomes Consortium 2016; Fulgione and Hancock 2018). Molecular analyses have shown that the development of trichomes in pedicels and stems of these populations are caused by *TCL1*. However, the production of trichomes in fruits requires loss-of-function alleles in the trichome repressors *TCL1* and *TRY*, and a gain-of-function allele in the *GL1* activator (Arteaga et al. 2021). This genetic analysis has also identified *MALAMBRUNO 1* (*MAU1*) as another locus affecting fruit trichome pattern, but its relevance on the evolution of this trait remains elusive.

In this study, we aim to isolate the gene underlying *MAU1* and to characterize its contribution to the natural variation for trichome development in the epidermis of different organs, and for other pleiotropic-related traits. Genetic and molecular analyses of introgression and transgenic lines carrying different *MAU1* natural alleles demonstrated that *EGL3* is *MAU1*. Additional phylogenetic analyses indicate that the evolution of trichomes in fruits of Arabidopsis is explained by the synergistic interactions between natural *EGL3* gain-of-function alleles and specific alleles of *TCL1*, *TRY*, and *GL1*. Finally, we show that *EGL3* also contributes to the natural variation for trichome branching, but not for anthocyanin epidermal content, thus revealing that different *EGL3* natural alleles display distinct pleiotropy and interactions in the underlying gene regulatory network.

## Results

### *MAU1* shows strong epistatic effect on fruit trichome pattern

**Figure 1.**
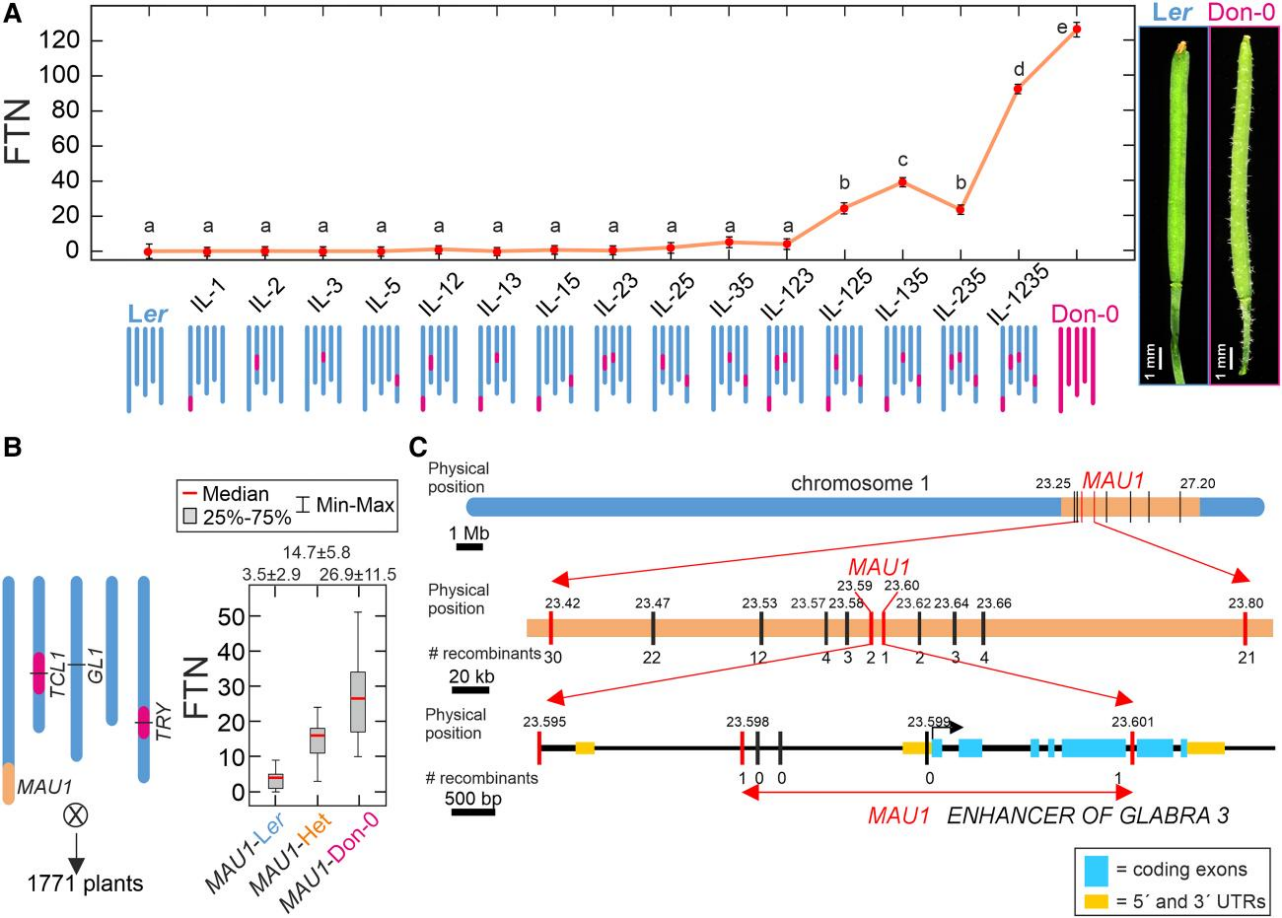
Genetic characterization and fine mapping of *MAU1*. **A)** Fruit trichome number (FTN) of parental and introgression lines (ILs) differing in Don-0/L*er* alleles at *MAU1*, *TCL1*, *GL1*, and *TRY* genomic regions. Dots and bars correspond to means ± 0.95 confidence intervals of two or three lines per genotype (8 to 18 plants per line). Representative fruits of parental accessions, Don-0 and L*er*, are shown in the right side, whereas graphical genotypes of ILs are depicted in the lower part of the panel. Phenotypic differences among genotypes were tested by mixed linear models, and the same or different letters indicate nonsignificant and significant differences, respectively, as estimated by Tukey's test (*P* < 0.05). **B)** Graphical genotype of the IL used for *MAU1* fine mapping showing the positions of *MAU1*, *TCL1*, *GL1*, and *TRY*; size of the mapping population; and FTN distributions of plants homozygous or heterozygous for Don-0 and L*er* alleles in *MAU1* mapping population. The average FTN values of 14 to 23 plants of each genotypic class are shown in the upper side of the panel. **C)** Fine mapping of *MAU1* showing the physical positions of markers and number of recombination events analyzed. In each panel, blue, magenta, and orange colors depict homozygous L*er*, homozygous Don-0, or Don-0/L*er* heterozygous genomic regions, respectively. UTR, untranslated region.

In contrast to the laboratory strain Landsberg *erecta* (L*er*) and most worldwide accessions, ∼ 10% of Arabidopsis Iberian populations, such as Doñana (Don-0), develop trichomes in fruits. Previous genetic analyses of the cross Don-0 × L*er* have shown that, in addition to the already identified genes *TCL1*, *GL1*, and *TRY*, the locus *MAU1* located at the bottom of chromosome 1 contributes to this trait (Arteaga et al. 2021). To characterize *MAU1* effects on trichome pattern and its genetic interactions with *TCL1* (middle of chromosome 2), *GL1* (middle of chromosome 3), and *TRY* (lower arm of chromosome 5), we developed a set of 15 different introgression lines (ILs) corresponding to all combinations of Don-0 alleles in one to four of these genomic regions, in an otherwise L*er* genetic background (Fig. 1A). ILs were named with the numbers of chromosomes containing Don-0 introgressed loci. We analyzed these Don-0/L*er* ILs for the amount of trichomes in multiple organs, quantified as fruit trichome number (FTN), pedicel and stem trichome pattern (PTP, STP) measured as the number of fruit pedicels or stem internodes with trichomes, respectively, and leaf trichome density (LTD) (Fig. 1, Supplementary Fig. S1).

Analysis of FTN showed that ILs carrying Don-0 alleles only in *MAU1*, or in combination with Don-0 alleles in one of the other three loci, developed no, or very few, (<5) trichomes per fruit (Fig. 1A). Only ILs with Don-0 alleles in three loci (IL-125, IL-135, and IL-235), or four loci (IL-1235), showed high FTN values, which indicates that this trait requires *TRY*-Don-0 alleles in combination with Don-0 alleles in two out of the remaining three loci. The trigenic *MAU1* interactions MAU1*GL1*TRY and MAU1*TCL1*TRY accounted for 21.2% and 12.3% of FTN phenotypic variance, whereas the digenic interaction MAU1*TRY explained 14.6% (Fig. 1A, Supplementary Table S1). Therefore, *MAU1* appears as a major effect locus on fruit trichome development, but mainly through synergistic epistasis with Don-0/L*er* natural alleles in *TRY*, *GL1*, and *TCL1*.

Additional analyses of ILs for trichome pattern in pedicels, stems, and leaves, showed that these traits were mostly explained by *TCL1* and its genetic interactions with *GL1* (Supplementary Fig. S1 and Table S1), in agreement with previous results (Arteaga et al. 2021, 2022). Accordingly, in L*er* genetic background, *MAU1* showed only marginal effect on the amount of trichomes in organs other than fruits.

To identify the gene underlying *MAU1*, we first fine mapped it using an IL homozygous for Don-0 alleles in *TCL1* and *TRY*, but heterozygous Don-0/L*er* for *MAU1* genomic region (Fig. 1B). As expected from the genetic interactions described above, *MAU1* showed an average FTN effect of 23.4 trichomes, measured in the self-progeny of this line as the difference between Don-0 and L*er* homozygous genotypes. In addition, *MAU1* heterozygous plants displayed an intermediate phenotype between homozygous plants, which indicates *MAU1* semidominance in this genetic background (Fig. 1B). Genetic analysis of 51 recombinants selected from a mapping population of 1,771 plants, could locate *MAU1* in a small genomic region of 3 kb where the Col reference genome sequence predicts that there is only one open-reading frame, *AT1G63650* (Fig. 1C). Interestingly, this gene corresponded to *ENHANCER OF GLABRA3* (*EGL3*), which encodes a bHLH transcription factor previously shown to regulate trichome pattern in leaves of Arabidopsis (Zhang et al. 2003).

### An *EGL3* gain-of-function allele underlies Don-0 *MAU1* locus

**Figure 2.**
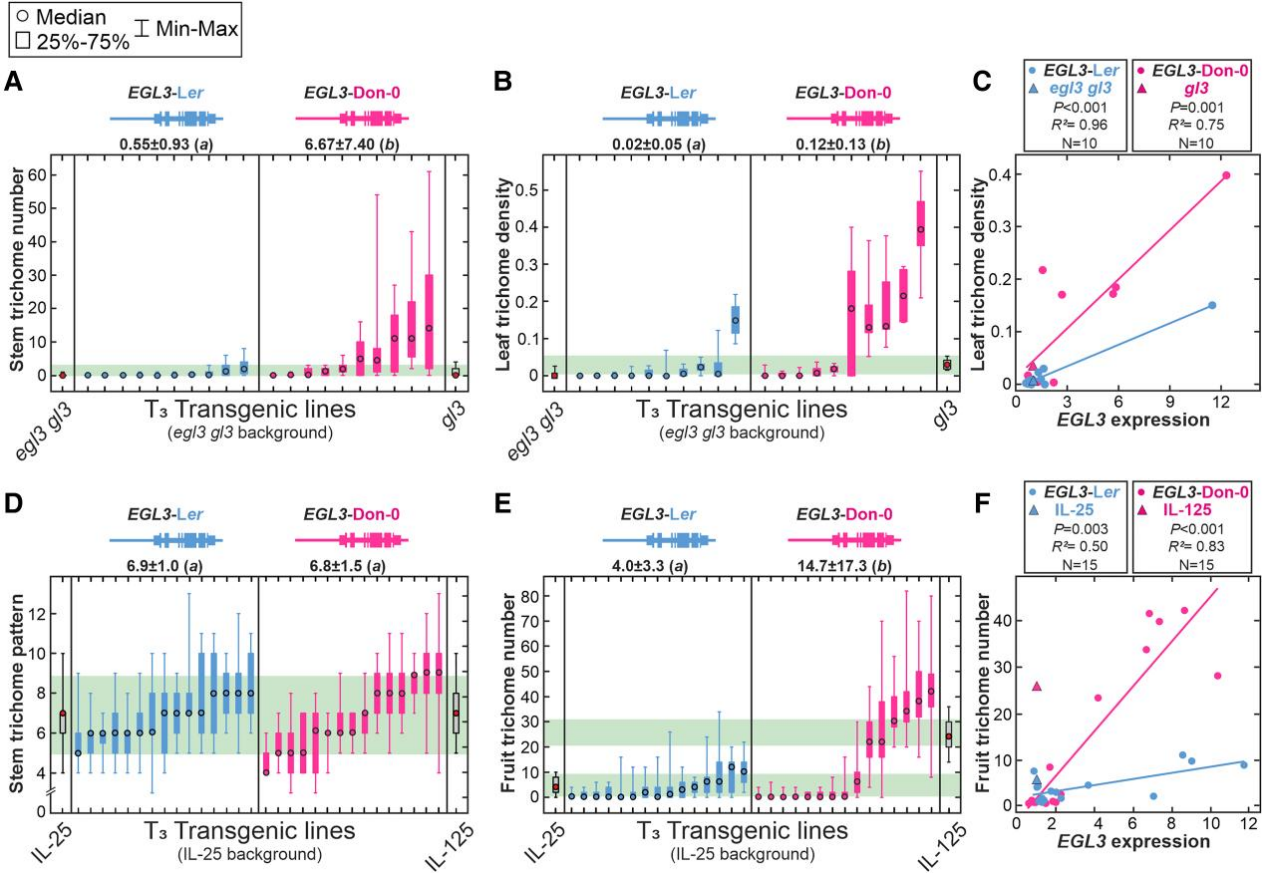
Trichome pattern and gene expression of *EGL3* transgenic lines. **A to E**) Stem (A, D), leaf (B), and fruit (E) trichome phenotypes of independent homozygous transgenic lines carrying L*er* or Don-0 genomic constructs of *EGL3*, in *egl3 gl3* mutant (A, B) or IL-25 (D, E) genetic backgrounds. **C, F**) Relationship between leaf (C) or fruit (F) trichome patterns and *EGL3* expression in leaves (C) or flower buds (F), for transgenic lines in *egl3 gl3* (C) or IL-25 (F) backgrounds. Gene expressions of transgenic lines are relative to the expression of untransformed controls. Stem trichome number (STN) was measured as the total number of trichomes (A), whereas stem trichome pattern (STP) is the number of hairy internodes in the main inflorescence (D). Leaf trichome density (LTD) is the number of trichomes per leaf mm^2^ (B), and fruit trichome number (FTN) is the number of trichomes in the first fruit (E). In A, B, D, and E, drawings of *EGL3* genomic constructs are shown on top of each panel, with magenta (Don-0) and blue (L*er*) colors depicting different coding and regulatory regions in both transgenes. In these panels, transgenic lines are arranged from low to high mean phenotypic values, and 95% confidence intervals for untransformed controls are shown as green-shaded areas. Phenotypic differences among genotypes were statistically tested by mixed linear models; the same or different letters on top of each panel indicate nonsignificant or significant differences, as tested by Tukey's test (*P* < 0.05). In **C** and **F**, linear regression analyses were used to test the relationships between *EGL3* expression and trichome traits.

In agreement with previous mutant analyses (Zhang et al. 2003), the artificial loss-of-function mutation *egl3* in L*er* genetic background showed a reduction of trichome number in leaves and stems (Supplementary Fig. S2) indicating that *EGL3* is a positive regulator of trichome development. This result suggested that, if *EGL3* is *MAU1*, Don-0 carries a gain-of-function allele. To evaluate this hypothesis, we first tested the functionality of *EGL3* Don-0 and L*er* alleles by developing two genomic constructs that include the promoter, coding, and 3′-UTR regions from each accession, and used them to generate transgenic lines in the *egl3 gl3* double mutant background. We chose this genotype because it displays a glabrous phenotype that reflects the partial redundancy previously described for *EGL3* and its homolog *GL3* (Zhang et al. 2003; Supplementary Fig. S2). Hence, such a glabrous phenotype might facilitate the qualitative detection of *egl3* genetic complementation by *EGL3* alleles from Don-0 and L*er*. Phenotypic characterization of 10 T_3_ homozygous independent transgenic lines generated for each construct showed that both natural *EGL3* alleles are functional because the two types of transgenic lines developed trichomes in leaves and stems of some lines (Fig. 2A-2B). On average, transgenic lines carrying *EGL3*-L*er* allele showed similar trichome pattern phenotypes than *gl3* mutant, thus evidencing complete *egl3* complementation. However, *EGL3*-Don-0 transgenic lines showed, on average, larger amount of trichomes in both organs (*P* < 0.05; Fig. 2A-2B, Supplementary Table S1) indicating that Don-0 carries an *EGL3* gain-of-function (or hypermorphic) allele compared with L*er*, which is able to overcome the lack of *GL3*.

To further evaluate if *EGL3* is *MAU1*, we also transformed the line IL-25 and quantified the effect of both transgenes on fruit, stem, and leaf trichomes (Fig. 2D-2E, Supplementary Fig. S3). This line was selected because, as described above, the effect of *MAU1* on FTN requires Don-0 alleles in at least *TRY* and *TCL1* loci. Taking into account that *EGL3* is a promoter of trichome development, it can then be expected that, in this background, *EGL3*-Don-0 and *EGL3*-L*er* transgenes will differentially increase FTN. Analysis of 15 T_3_ homozygous independent transgenic lines for each construct showed significant differences between *EGL3*-Don-0 and *EGL3*-L*er* alleles for FTN, similar to those observed between lines IL-25 and IL-125 (Fig. 2E). On average, *EGL3*-Don-0 transgenic lines had three times higher FTN values than *EGL3*-L*er* lines. However, as described for the lines IL-25 and IL-125, the two classes of transgenic lines did not differ in the trichome pattern of stems (Fig. 2D), or leaves (Supplementary Fig. S3). Overall, for each transgene, lines with more trichomes in fruits also showed higher density in leaves (*r* = 0.91 and 0.77 for *EGL3*-Don-0 and *EGL3*-L*er* transgenic lines, respectively). However, *EGL3*-Don-0 transgene displayed a significantly higher effect than *EGL3*-Ler only on FTN, but not on LTD (Supplementary Fig. S3B). Therefore, we concluded that *EGL3* underlies *MAU1*, for which Don-0 carries a gain-of-function allele specific for fruit trichome pattern in this genetic background.

### *EGL3* gain-of-function allele is not caused by prevalent *cis*-regulatory mutations

**Figure 3.**
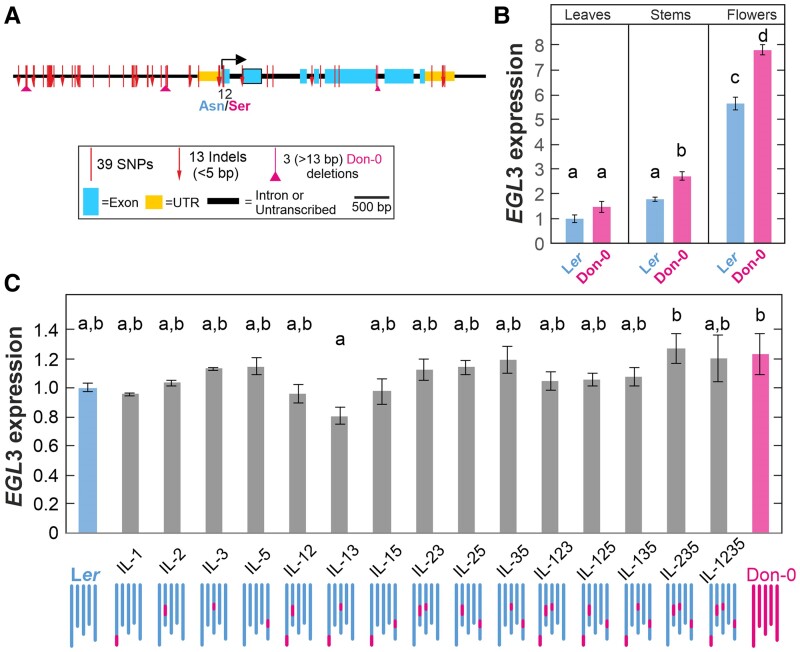
Nucleotide diversity and gene expression of *EGL3* in parental accessions and introgression lines. **A)** Genomic structure of *EGL3*, including the location and total number of SNPs and indels differentiating L*er* and Don-0 accessions. **B)**  *EGL3* expression in leaves, stems, and flower buds of Don-0 and L*er* accessions. **C)**  *EGL3* expression in flower buds of ILs bearing Don-0 alleles in *MAU1*, *TCL1*, *GL1*, and/or *TRY* regions. In **B** and **C**, each bar depicts the mean ± SE of three biological replicates, and values of all lines are relative to L*er* vegetative (B) or reproductive (C) expression. Statistical differences among genotypes were tested by mixed linear models, and the same or different letters indicate nonsignificant and significant differences, respectively, as estimated by Tukey's test (*P* < 0.05). UTR, untranslated region.

To determine if the gain of function of *EGL3*-Don-0 allele is caused by *cis*-regulatory mutations affecting gene expression, or by protein structural mutations, we first compared *EGL3* sequences in parental accessions Don-0 and L*er.* A total of 55 nucleotide polymorphisms differentiated both parents, with most *EGL3* single-nucleotide polymorphisms (SNPs) and indels locating in the promoter region (Fig. 3A). In addition, one missense mutation (Asn to Ser) was found affecting the amino acid at position 12 within the MYC N-terminal domain of the protein. Since both types of polymorphisms might affect *EGL3* function we also compared gene expression levels in the parents, the ILs and the transgenic lines carrying *EGL3*-Don-0 and *EGL3*-L*er* alleles. Expression analyses of parental accessions in different organs showed that *EGL3* displays ∼ 2- to 5-fold higher expression in stems and flower buds than in leaves (Fig. 3B). In addition, Don-0 showed a weak but significantly higher *EGL3* expression than L*er* in stems and flowers, but not in leaves (*P* < 0.01), which indicates the occurrence of Don-0/L*er* polymorphisms affecting *EGL3 trans*- and/or *cis*-regulation. Further analyses of *EGL3* expression in flower buds of the 15 Don-0/L*er* ILs showed that most lines did not differ significantly (Fig. 3C). Overall, only line IL-235 displayed a high *EGL3* expression similar to Don-0 accession and significantly higher than IL-13. Statistical analysis of the four genomic regions segregating in the ILs showed that *TCL1* and *EGL3*/*MAU1* affected significantly *EGL3* expression, thus revealing polymorphisms affecting both, *trans*- (in *TCL1*) and *cis*- (in *EGL3/MAU1*), regulations (Supplementary Table S1). However, contrary to *EGL3 trans*-regulation mediated by *TCL1*, Don-0 allele of *EGL3* showed opposite effect than expected from the expression of Don-0 parental accession because it weakly reduced *EGL3* expression. Hence, *cis*-regulatory mutations accounting for such decrease of *EGL3* expression cannot explain the increased trichome densities caused by *EGL3*-Don-0 gain of function.

To validate the contribution of *EGL3 cis*-regulatory polymorphisms, we also analyzed *EGL3* expression in leaves and flower buds of the 50 transgenic lines generated in *egl3 gl3* and IL-25 genetic backgrounds. Independent lines carrying the same transgene showed up to 10-fold variation in *EGL3* expression in both backgrounds (Fig. 2C and F, Supplementary Table S1). Overall, lines with higher *EGL3* expression displayed higher trichome density in fruits (IL-25 background; Fig. 2F) and leaves (*egl3 gl3* background; Fig. 2C), thus indicating that both *EGL3* natural alleles encode functional proteins that positively regulate trichome development. In addition, artificial *EGL3* expression differences caused by the different genome positions of independent transgene insertions, largely account for the phenotypic variation observed among lines of the same transgene. However, in contrast to the significant phenotypic differences detected between the two transgenes, on average, *EGL3*-Don-0 and *EGL3*-L*er* transgenic lines did not differ in *EGL3* expression levels, in any background (*P* > 0.45; Supplementary Table S1). This result indicates that Don-0 and L*er* transgenes, carrying their own native promoters and untranslated regions, do not differ in *cis*-regulatory mutations affecting the general *EGL3* expression. Therefore, the natural gain-of-function *EGL3*-Don-0 allele that increases the amount of trichomes is likely caused by the effect of Asn/Ser^12^ substitution on EGL3 protein structure and function.

### *EGL3* contributes to the natural variation for trichome branching but not for anthocyanin content

**Figure 4.**
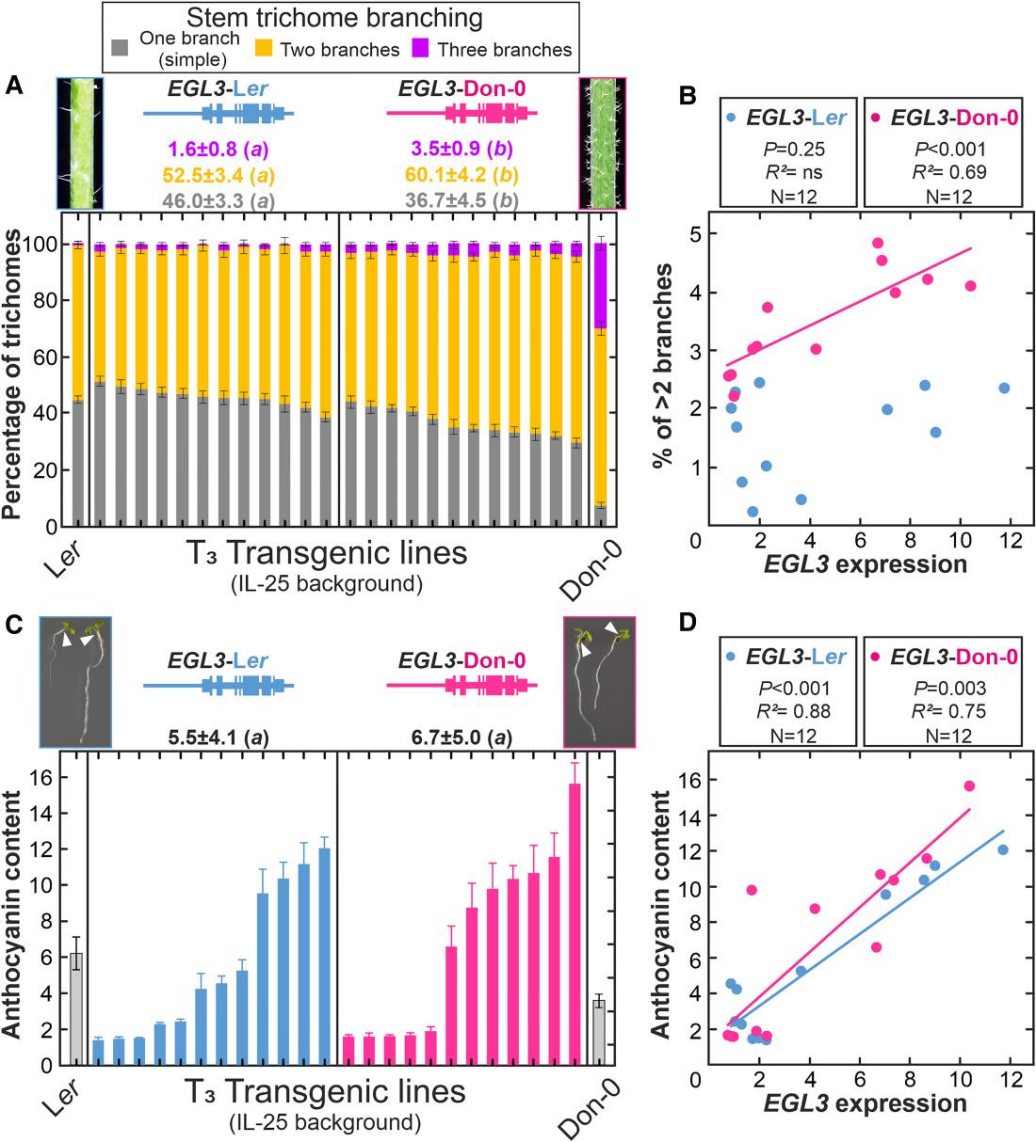
Stem trichome branching and hypocotyl anthocyanin content of *EGL3* transgenic lines. **A, C)** Frequency of stem trichomes with one, two, or more than two branches (A), or hypocotyl anthocyanin content (C) in independent homozygous transgenic lines carrying L*er* or Don-0 genomic constructs of *EGL3* in IL-25 genetic background. The bar of each transgenic line shows the percentage ± SE of trichomes of each branching class quantified in 10 to 18 plants per line (A) or the mean ± SE anthocyanin content measured in three replicates of 30 seedlings (C). Transgenic lines are arranged from low to high mean phenotypic values. Anthocyanin content was measured as units of spectrophotometer absorbance per mg of fresh weight. The average phenotypes of the lines for each transgene, and pictures of parental lines, are shown on top of the panels. White arrowheads in seedling pictures of panel C point to the hypocotyl anthocyanin. Phenotypic differences among genotypes were statistically tested by mixed linear models; the same or different letters on top of each panel indicate nonsignificant or significant differences, as tested by Tukey's test (*P* < 0.05). **B, D)** Relationship between the frequency of trichomes with more than two branches (B) or the anthocyanin content (D), and *EGL3* expression in flower buds. Linear regression analyses were used to test the relationships between *EGL3* expression and trichome or anthocyanin traits.

Since previous studies of artificial *egl3* mutants have shown that *EGL3* affects pleiotropically other traits, such as trichome branching and anthocyanin content (Zhang et al. 2003; Supplementary Fig. S2), we further analyzed the effects of natural Don-0/L*er* alleles of *EGL3* on these phenotypes. In particular, in contrast to Arabidopsis laboratory L*er* strain characterized by stems with mainly trichomes of one (simple) or two branches (>99%), Don-0 stems mostly develop branched trichomes (>90%) with an average of 30.2% having three or more branches (Fig. 4A). Phenotypic analysis of 24 independent transgenic lines homozygous for *EGL3*-Don-0 or *EGL3*-L*er* alleles in IL-25 background showed that *EGL3* accounts for 11% to 21% of the variance for trichomes with one, two, or more than two branches (Supplementary Table S1). On average, lines carrying *EGL3*-Don-0 transgene developed significantly higher proportion of trichomes with two, three or more branches, as well as lower amount of simple trichomes than *EGL3*-L*er* lines (Fig. 4A; Supplementary Table S1). In addition, in *EGL3*-Don-0 transgenic lines, the amount of *EGL3* expression correlated positively with the proportion of highly branched trichomes, but this was not the case for *EGL3*-L*er* lines (Fig. 4B). Therefore, *EGL3*-Don-0 appears as a strong positive regulator of trichome branching, whereas *EGL3*-L*er* shows smaller effect, in accordance with the weak phenotypes of mutant alleles in L*er* background (Supplementary Fig. S2; Zhang et al. 2003).

To test the effect of natural Don-0/L*er* alleles of *EGL3* on anthocyanin content, we further characterized the same 24 *EGL3* transgenic lines for the amount of pigment in hypocotyls (Fig. 4C). Although independent lines of each transgene showed substantial variation for anthocyanin content, on average, transgenic lines with *EGL3*-Don-0 and *EGL3*-L*er* alleles did not differ significantly for this trait (Fig. 4C; Supplementary Table S1). However, *EGL3* expression accounted for 75% to 88% of the variance for anthocyanin content among lines, as shown by the significant positive correlation found between the two variables in both, *EGL3*-Don-0 and *EGL3*-L*er*, transgenic lines (Fig. 4D). Hence, in agreement with the behavior of loss-of-function mutants (Supplementary Fig. S2), *EGL3* is a positive activator of anthocyanin content, but Don-0 and L*er* alleles do not differ in their pleiotropic effects on this trait.

### Multiple independent *EGL3* gain-of-function alleles explain fruit trichome development in Arabidopsis

**Figure 5.**
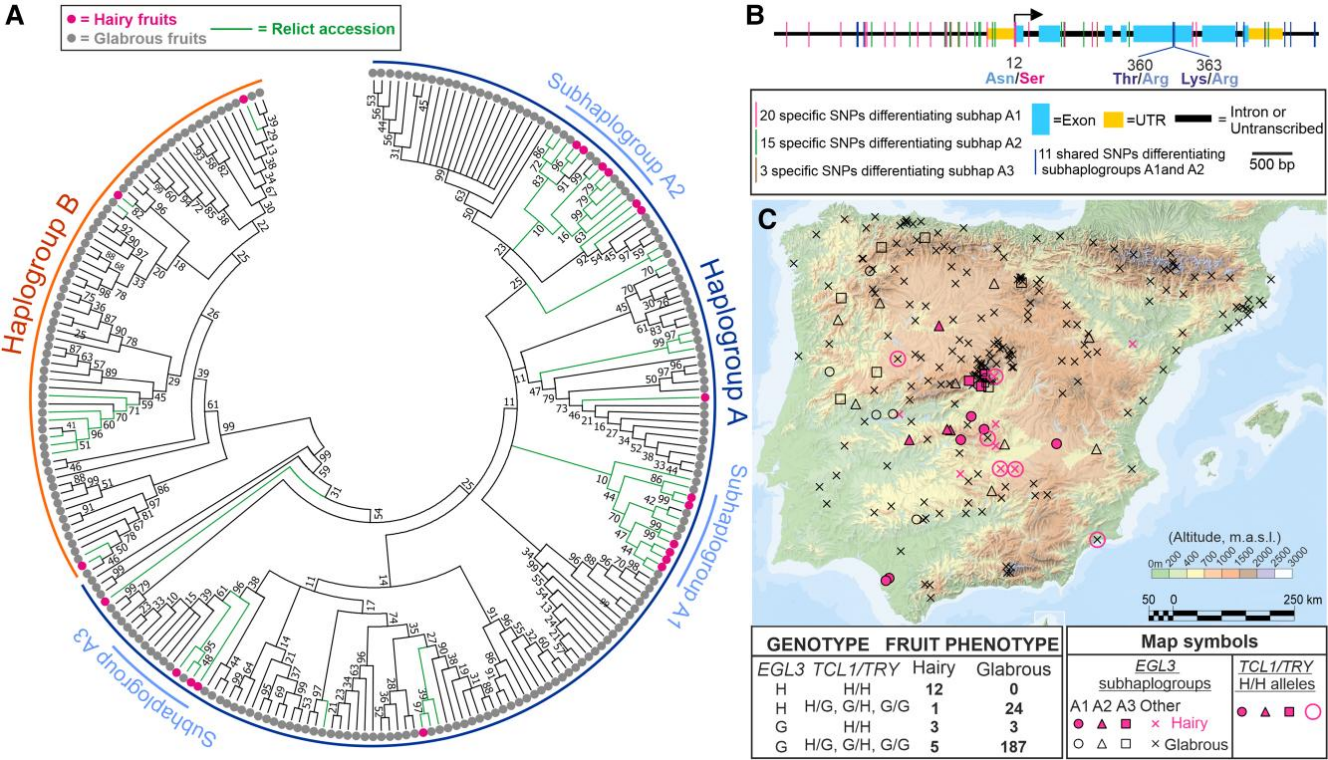
Nucleotidic and geographic diversity of *EGL3*. **A)** Topology of neighbor-joining tree displaying the genetic relationships among 235 Iberian accessions for *EGL3*. Branches corresponding to partitions reproduced in <10% bootstrap replicates are collapsed, and branches corresponding to relict accessions are colored in green. Clusters of accessions corresponding to the major haplogroups A and B, and to the subhaplogroups A1, A2, and A3, are highlighted with colored lines. **B)** Genomic location of SNPs differentiating the A1, A2, and A3 subhaplogroups of *EGL3*. Missense mutations segregating in different subhaplogroups are indicated with different colors. **C)** Geographic distribution of Iberian populations classified according to the qualitative trichome pattern of fruits (hairy or glabrous), and their alleles in *EGL3*, *TCL1*, and *TRY*. Map symbols depicting populations with different *EGL3* genotypes and phenotypes are described in the legend. Table below the map shows the number of accessions with different genotypes and phenotypes. For this, accessions carrying *EGL3* subhaplogroups A1, A2, or A3 are classified as H, whereas those with other haplotypes are genotyped as G. For *TCL1* and *TRY*, accessions are classified based on the natural polymorphisms previously described to reduce gene functions (Arteaga et al. 2021): H, when they carry partial loss-of-function alleles causing the hairy phenotype; G, when bearing functional glabrous alleles.

To further understand the contribution of *EGL3* to the natural diversity for fruit trichome pattern, we investigated its nucleotidic and geographic diversity. Analysis of *EGL3* genomic sequences from 235 accessions of the Iberian Peninsula showed that most nucleotide diversity concentrates in two promoter regions, as well as in exons 5 and 6 (Supplementary Fig. S4A). Phylogenetic analysis of these sequences identified two major haplogroups, named as A (70.2%) and B (29.8%), which are differentiated by 38 SNPs, mostly located in exons 5 and 6 (Fig. 5A). These polymorphisms included four missense mutations that did not affect highly conserved protein domains, although one of them (Val/Ile^328^ mutation; Supplementary Fig. S4C), appeared as completely conserved in EGL3 proteins from Brassicacea. In addition, for two of these missense mutations, the two amino acids segregating at Arabidopsis intraspecific level also segregated among Brassicaceae species, thus suggesting an old origin of such mutations in the common Brassicaceae ancestor. The two *EGL3* haplogroups displayed a worldwide geographic distribution, with similar frequencies of the minor haplogroup B in Eurasia and Africa, but a lower representation in North America (Supplementary Fig. S4B). Furthermore, both haplogroups also segregated within the two major genetic lineages previously described as relict and nonrelict in Arabidopsis, which separated ∼ 60 kya (1001 Genomes Consortium 2016). By contrast, the accessions developing trichomes in fruits belong to the relict group from the Iberian Peninsula (Fig. 5A; Arteaga et al. 2021), either from haplogroup A (86%) or B (14%) (Supplementary Fig. S4A). Therefore, the nucleotide diversity differentiating the two main *EGL3* haplogroups likely originated earlier in Arabidopsis history than the separation of relict and nonrelict lineages, and it is not significantly associated with fruit trichome pattern variation.

Analysis of the 21 Iberian accessions known to develop trichomes in fruits, (Arteaga et al. 2021) identified no *EGL3* haplotype shared by most of them, which indicates that a single *EGL3* gain-of-function allele did not account for all accessions with this trait. However, hairy fruit accessions were not evenly distributed across *EGL3* nucleotide diversity, but 13 (62%) of them, all belonging to haplogroup A, clustered within three distinct *EGL3* haplotypes named as subhaplogroups A1, A2, and A3 (Fig. 5A). This enrichment of hairy fruit accessions in three subhaplogroups suggested the occurrence of, at least, three independent *EGL3* gain-of-function alleles of low frequency. Analysis of polymorphisms in *EGL3* coding region showed only three missense mutations segregating in subhaplogroups A1 and A2, but these mutations were not shared by all hairy accessions of each subhaplogroup (Fig. 5B). One of these polymorphisms was Don-0 Asn/Ser^12^ missense mutation present in two out of six hairy accessions of subhaplogroup A1, which indicates that only another accession carries also Don-0 gain-of-function allele (Fig. 5A). In addition, subhaplogroups A1, A2, and A3 were differentiated by other 19, 13, and 3 specific SNPs, respectively, mostly located in *EGL3* promoter (Fig. 5B). Overall, the low frequency of *EGL3* polymorphisms hampered the inference of additional mutations that might cause *EGL3* gain-of-function. However, this pattern of nucleotide diversity suggests that multiple polymorphisms might account for the gain-of-function of each subhaplogroup, and that both, structural and *cis*-regulatory, mutations likely contribute. Furthermore, analysis of these SNPs in the Eurasian and African genome sequences currently available (1001 Genomes Consortium 2016; Durvasula et al. 2017) indicates that these haplotypes originated in this geographic region because all polymorphisms are specific of the Iberian Peninsula.

To assess the relevance of these potential *EGL3* gain-of-function alleles, we also analyzed their effect on fruit trichome development. Given the strong genetic interactions found between *EGL3*, *TCL1*, and *TRY*, the effect of the three genes was simultaneously considered. *GL1* was not included in this analysis because its Don-0 gain-of-function allele has only been found in two accessions (Arteaga et al. 2021; Supplementary Table S2). To this end, we qualitatively classified the 235 Iberian accessions as hairy (H) and glabrous (G) according to the presence of trichomes in fruits (Fig. 5C). Similarly, accessions were genotyped as H for *EGL3* when they belong to potential *EGL3* gain-of-function subhaplogroups A1, A2, and A3, independently of the polymorphisms differentiating them. Likewise, populations were genotyped as H for *TCL1* or *TRY* when they bear the partial loss-of-function mutations *TCL1* 168 bp deletion or *TRY* SNP-701, previously described as necessary for fruit trichome development (Supplementary Table S2). This genotyping showed that all but one of the hairy fruit accessions belonging to *EGL3* subhaplogroups A1, A2, or A3, also carried H alleles in *TCL1* and *TRY* genes (Fig. 5C). On the contrary, all 24 glabrous fruit accessions belonging to *EGL3* subhaplogroups A1, A2, or A3, had common functional glabrous alleles of *TCL1* and/or *TRY* genes. Accordingly, statistical analysis detected no additive effect of *EGL3* on the hairy fruit phenotype (*P* = 1; Supplementary Table S1). However, the trigenic interaction between *EGL3*, *TCL1*, and *TRY* showed the strongest effect (*P* < 10^−5^; explained variance = 30.6%) followed by *TCL1*, *TRY*, and their digenic interaction (*P* < 0.002; Supplementary Table S1). In addition, hairy fruit populations of *EGL3* subhaplogroups showed more restricted geographic distribution (maximum pair-wise distances among populations of 463, 239, and 35 km for subhaplogrups A1, A2, and A3) than the glabrous fruit populations with the same *EGL3* haplogroups (maximum distances of 512, 603, and 446 km, respectively) (Fig. 5C). Therefore, the development of trichomes in fruits is largely explained by several *EGL3* gain-of-function alleles that interact with loss-of-function mutations in *TCL1* and *TRY* mostly distributed in the relict lineage of southern Iberia.

## Discussion

### Synergistic interactions between *EGL3* gain-of-function alleles and MYB genes account for fruit trichome development

**Figure 6.**
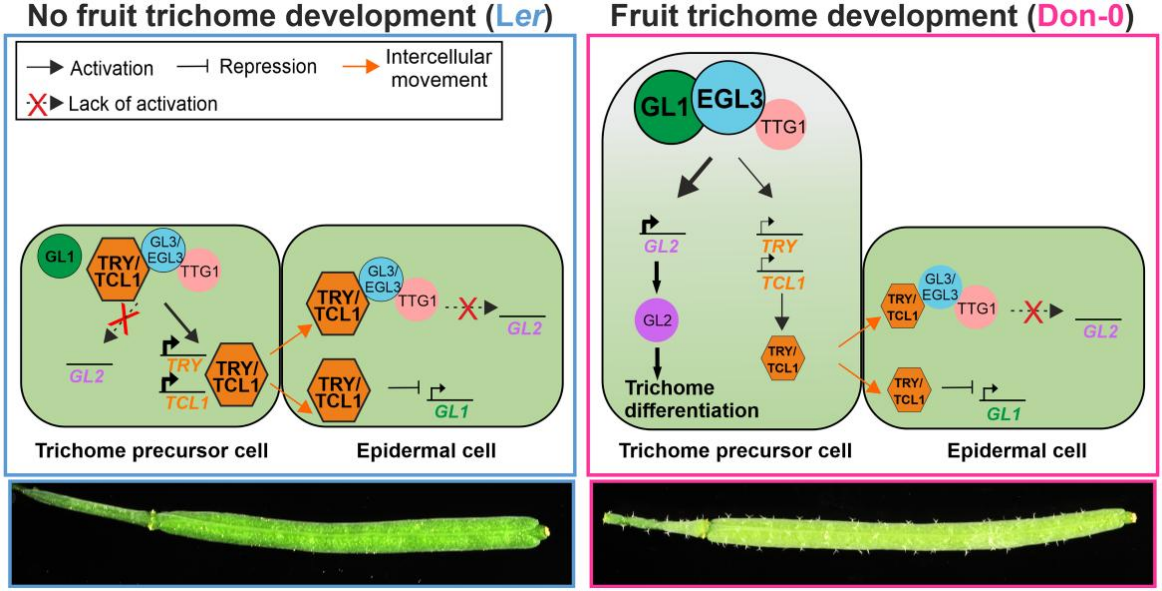
Molecular model for the natural variation in the regulation of fruit trichome development in Arabidopsis. Panels depict the core gene regulatory network of trichome initiation in epidermal precursor and neighboring cells (reviewed in Pattanaik et al. 2014; Wang and Chen 2014) of wild accessions with (right panel), or without (left panel) trichomes in fruits. Transcription factors in the core regulatory network known to contribute to the natural variation for fruit trichome patterning are shown, including the component identified in this study, *EGL3*, and those previously found, *TCL1*, *TRY,* and *GL1* (Arteaga et al. 2021). The molecular mechanisms underlying natural alleles of these genes are represented by the size of protein symbols and transcriptional arrows, larger sizes depicting increased protein activity (*EGL3*, *GL1*) or increased amount (*TCL1* and *TRY*).

The development of trichomes in fruits is presumably an adaptive qualitative trait that has evolved in multiple species from most tribes of Brassicaceae, and that is used as taxonomic trait also in many other Angiosperm families (Tutin et al. 1993). In this study, we identified the bHLH transcription factor gene *EGL3*, as a molecular component underlying the *MAU1* locus to account for this evolutionary innovation in the Arabidopsis relict lineage of the Iberian Peninsula (Arteaga et al. 2021). Two main genetic features of *EGL3*, such as the occurrence of natural gain-of-function alleles and its synergistic epistasis with multiple MYB genes, determine the evolution of trichome formation in carpels and fruits of Don-0 accession. Phenotypic and molecular characterization of transgenic lines in two different genetic backgrounds demonstrate that *EGL3*-Don-0 is a gain-of-function allele since it increases trichome density in comparison to *EGL3*-L*er* transgene. As expected, this gain-of-function allele behaves as semidominant, which likely facilitates its selection and maintenance in nature. However, *EGL3* gain-of-function is necessary but not sufficient to evolve this trait because, as shown with the analysis of introgression lines, it also requires the combination of specific natural alleles in at least three loci, *EGL3* and *TRY*, as well as *TCL1* or *GL1*. Therefore, the development of fruit trichomes in Don-0 accession is caused by the synergistic interaction between two gain-of-function alleles of *EGL3* and the R2R3 MYB gene *GL1*, and two partial loss-of-function alleles of the R3 MYB genes *TRY* and *TCL1* (Fig. 6). Although we could not prove the polymorphism(s) causing *EGL3*-Don-0 gain of function, the lack of expression differences between Don-0 and L*er* transgenic lines suggests that *EGL3* hyperfunction and dominance is explained by the missense mutation Asn/Ser^12^ affecting the MYC N-terminal domain. In agreement with this, Asn^12^ is a highly conserved residue in most EGL3 proteins from Brassicaceae since only one in 23 species was different (Supplementary Fig. S4). In addition, the EGL3 MYC amino domain has previously been involved in protein interactions with GL1, and Ser^12^ mutation could thus affect the formation and function of the trimeric complex that activates trichome differentiation (Zhang et al. 2003; Zhang and Hülskamp 2019). Nevertheless, we cannot exclude that other structural mutations might also be necessary for the gain-of-function of *EGL3*-Don-0, such as those differentiating the two major *EGL3* haplogroups detected in this study. Alternatively, other regulatory mutations affecting the precise temporal or cellular *EGL3* expression could also contribute, because artificial differences in *EGL3* levels showed strong phenotypic effects in transgenic lines.

Furthermore, several independent *EGL3* gain-of-function alleles seem to account for the evolution of hairy fruits in relict populations other than Don-0, as suggested by the clustering of most accessions with this phenotype in three subhaplogroups A1, A2, and A3 detected by phylogenetic analyses. In contrast to the two highly differentiated *EGL3* haplogroups A and B, which likely diverged before the preglacial separation of relict and nonrelict accessions (1001 Genomes Consortium 2016; Fulgione and Hancock 2018), these hairy *EGL3* subhaplogroups probably have a recent origin in this region, as pointed by the smaller number of polymorphisms differentiating them. Missense mutations likely cause the gain-of-function of some of these alleles, since the Asn/Ser^12^ change was also present in another accession carrying a very similar *EGL3*-Don-0 allele, and two other amino acid substitutions segregated in two of these *EGL3* subhaplogroups (Fig. 5B). However, additional regulatory mutations must also contribute to the potential *EGL3* gain of function of some accessions, because missense mutations did not affect to all hairy fruit accessions. The occurrence of several *EGL3* gain-of-function alleles was also supported by the strong synergistic interaction detected in Iberian accessions between *EGL3* and functional mutations previously described for *TRY* and *TCL1* genes. In agreement with the epistasis found in ILs, this trigenic interaction accounted for 12 (57%) of the hairy fruit accessions. However, in contrast to the multiple gain-of-function alleles with low frequency speculated for *EGL3*, a single loss-of-function allele of *TRY* and *TCL1* occurs in most relict accessions with hairy fruits. In addition, the gain-of-function allele *GL1*-Don-0 has only been found in the same two Iberian accessions carrying *EGL3*-Don-0 allele (Arteaga et al. 2021). We therefore hypothesized that the evolution of trichomes in fruits of Arabidopsis has occurred by the combination of standing mutations in *TRY* and *TCL1* present in southern Iberia relict populations, with new *EGL3* and *GL1* gain-of-function alleles arisen more recently in this genetic lineage. In agreement with a wealth of theoretical literature (Phillips 2008; Ågren 2021; Li and Bank 2024), these results provide experimental evidence on the relevance of hyperfunctional dominant alleles and complex multigenic epistasis for the evolution of novel adaptive traits in plants. Nevertheless, additional causal mutations in these and/or other genes await identification to fully explain the genetic mechanisms underlying the evolution of hairy fruits in Arabidopsis.

### *EGL3* interactions in the trichome regulatory network explain the diversity of pleiotropic effects on epidermal traits

The development of trichomes in Arabidopsis fruits involves the activation of the trichome developmental program in a specific flower whorl organ, such as the ovary of the carpel. Previous studies have shown that the bHLH transcription factor EGL3 occupies a central position in the core gene network regulating trichome development because it is a component of the trimeric complex that activates this developmental program (Zhang et al. 2003; Morohashi and Grotewold 2009; Doroshkov et al. 2019; Zhang and Hülskamp 2019). As part of this complex, EGL3 physically interacts with the R2R3 MYB GL1 transcription factor to activate trichome development, but it also binds to competing R3 MYB proteins that lack the transcriptional activation domain to inactivate the trimeric complex (Pattanaik et al. 2014; Wang and Chen 2014; Zhang and Hülskamp 2019). Our results indicate that the *EGL3* gain-of-function allele mutated in Ser^12^ increases the function of this activation complex. However, the strong synergistic interactions described above further evidence that two other components of the trichome gene regulatory network need to be mutated to evolve trichome development in carpels and fruits (Fig. 6). First, the function of the activation complex is further increased by another *GL1* gain-of-function mutation. Numerous amino acid substitutions characterize *EGL3* and *GL1* diversity (Supplementary Fig. S4A and Arteaga et al. 2021), which suggests that the function of this complex has largely evolved in Arabidopsis through structural mutations in both proteins. Second, in hairy fruit accessions, the amount of R3 MYB transcription factors that repress the activation complex is strongly reduced in reproductive tissues, which is achieved by means of partial loss-of-function regulatory mutations in *TCL1* and *TRY* (Arteaga et al. 2021, 2022). Thus, changing the amount and extent of R3 MYB gene expression appears as another necessary mechanism to modify the trichome pattern in specific organs (Fig. 6). In agreement with these multigenic interactions, the artificial overexpression of *EGL3* or *GL1* was not sufficient to develop trichomes in Arabidopsis fruits (Zhang et al. 2003), but it required overexpressing *EGL3* or *GL1*, in combination with artificial knockdown of *TRY*, or overexpression of the *GL1* paralogue *WER* (Ó'Maoiléidigh et al. 2013, 2018; Song et al. 2015). Therefore, the evolution of fruit trichome development has required mutations in multiple components of the core gene regulatory network.

In addition to trichome pattern, *EGL3* has been shown to display pleiotropic effects also on trichome branching, as well as on anthocyanin content, root hair formation, and seed coat color (Zhang et al. 2003; Zhang and Hülskamp 2019). Such pleiotropy has mainly been explained through a combinatorial model of three bHLH and eight R2R3 MYB transcription factors to produce different trimeric complexes that trigger specific epidermal programs. In this context of modular pleiotropy (Wagner and Zhang 2011), the two paralogous bHLH transcription factors *EGL3* and *GL3* display substantial pleiotropy because they interact with numerous MYB proteins. However, the particular properties of *EGL3* and *GL3* further account for their partial redundant functions, since *EGL3* and *GL3* have been mainly involved, respectively, in anthocyanin and trichome regulation (Supplementary Fig. S2; Zhang et al. 2003; Morohashi et al. 2007; Zhang and Hülskamp 2019). In agreement with this, we have shown that natural alleles of *EGL3* not only interact genetically with MYB genes, but also with the bHLH gene *GL3* to regulate trichome pattern in other organs than fruits, such as stems and leaves. In particular, our results suggest that *EGL3*-Don-0 is a gain-of-function allele for the amount of trichomes of leaves and stems only in the absence of functional *GL3* because these traits were affected in *egl3 gl3* transgenic lines, but not in IL-25 genetic background with an active *GL3* allele. *GL3* thus appears as involved in trichome development mainly in leaves and stems, whereas phenotypic and gene expression analyses indicate that *EGL3* affects trichome pattern specially in flower organs. Furthermore, in contrast to previously characterized *EGL3* alleles (Zhang et al. 2003; Lloyd et al. 2017), the gain-of-function allele *EGL3*-Don-0 also explained Arabidopsis natural variation for trichome branching, but not for anthocyanin content in hypocotyls. These results indicate that the precise pleiotropy of *EGL3* depends on its natural allele, which might be modified through the physical interactions with MYB transcription factors of the trimeric complex, likely mediated by *EGL3*-Don-0 Ser^12^ mutation. Therefore, the evolutionary subfunctionalization of duplicated genes, such as *EGL3* and *GL3*, appears as a continuous dynamic process that changes gene pleiotropy, and caution should be taken when inferring gene functional differentiations based on the characterization of single natural alleles.

### *EGL3* is an Arabidopsis target for adaptive evolution of trichome pattern and morphology

Our study identifies *EGL3* as a gene accounting for the natural diversity for trichome pattern and branching in reproductive organs. Another bHLH gene, *MYC1*, has been previously found as explaining the natural variation for Arabidopsis leaf trichome pattern (Symonds et al. 2011), although it is not part of the central trimeric complex that activates trichome development, but a downstream activator (Morohashi and Grotewold 2009; Zhang and Hülskamp 2019). By contrast, despite bHLH transcription factors have been involved in the regulation of trichome patterning in numerous crop species, such as cucumber, cotton, tomato, or maize (Zhang and Hülskamp 2019; Chalvin et al. 2020; Han et al. 2022; Feng et al. 2023) their contribution to trichome diversity in these species remains unknown. Furthermore, only *EGL3* and *TCL1* have been proven to account for the natural diversity of trichome branching in Arabidopsis (this study and Arteaga et al. 2022), thus showing the relevance of the core regulatory network of trichome development also for the evolution of trichome morphology in Brassicaceae. The molecular mechanisms underlying the natural variation for trichome size are just beginning to be deciphered also in several plants with unbranched trichomes, as shown with the recent identification of *HL6* in rice, *H* in tomato, *SPINE BASE SIZE* (*SBS1*) in cucumber, and *GUIDELESS* in monkeyflower (Shang et al. 2020; Yang et al. 2022a; Gasparini et al. 2023; Chen and Yuan 2024). These genes encode HD, R2R3 MYB, and C2H2 transcription factors, which are similar to the main regulators of trichome pattern. Therefore, the same gene regulatory network seems to control trichome pattern and morphology in different plant families, but distinct mechanisms might explain the natural diversity for both trichome components in each species.

It is likely that Arabidopsis *EGL3* diversity described here is involved in climatic adaptation because previous studies have shown that the development of trichomes in Arabidopsis fruits and other organs is associated with low spring precipitation (Arteaga et al. 2021, 2022). However, the precise ecological mechanisms maintaining trichome diversity in nature remain a challenging question because it depends on the particular trichome trait, plant organ, or plant species. Alike Arabidopsis, in *Quercus* and *Mimulus guttatus*, high leaf trichome density has been linked to climatic factors such as high temperatures and UV radiation, thus suggesting protection against water-loss or drought (Kooyers et al. 2015; Mediavilla et al. 2019). On the contrary, in other wild and crop plants, such as *Antirrhinum*, *Cardamine hirsuta*, or soybean, high trichome density in leaves and stems has been associated with opposite climatic variables, like low temperature or precipitation (Liu et al. 2020; Tan et al. 2020; Fuster-Pons et al. 2024). Additional studies in numerous plants have shown that the natural qualitative variation for hairy and glabrous populations is involved in plant defence against herbivorous insects (Mauricio and Rausher 1997; Kivimäki et al. 2007; Sato et al. 2019; Xuan et al. 2020). Similarly, the natural diversity for trichome morphology in fruits of cucumber and flowers of monkeyflower has been associated with different environmental factors, such as resistance to aphid feeding and flower–pollinator interactions (Chen and Yuan 2024; Yang et al. 2024). Hence, future comparative studies addressing trichome natural diversity are required to decipher the parallelism and divergence in the ecological and molecular mechanisms driving the adaptive evolution of trichome pattern, trichome morphology, and other pleiotropic epidermal traits of Angiosperms.

## Materials and methods

### Plant material, introgression lines, and fine mapping

The wild accessions of Arabidopsis (*Arabidopsis thaliana*) Don-0 and L*er* analyzed in this study were originally collected in Spain and Poland, respectively, and have previously been described (Alcazar et al. 2014; Arteaga et al. 2021).

A population of 43 ILs carrying Don-0 alleles at *MAU1* located at the bottom of chromosome 1, and the regions around *TCL1* (in the middle of chromosome 2), *GL1* (middle of chromosome 3), or *TRY* (lower arm of chromosome 5), in a L*er* genetic background, was analyzed. Twenty-one of these ILs carrying Don-0 alleles at *TCL1*, *GL1*, or *TRY*, have been described before (Arteaga et al. 2021) and correspond to three independent lines for each of the seven different combinations of one to three of those genomic regions from Don-0. Twenty-two additional lines carrying Don-0 alleles at *MAU1*, *TCL1*, *GL1*, or *TRY* were developed in this study. These additional ILs correspond to two or three independent lines for each of the eight different combinations of Don-0 alleles in the target locus of this study, *MAU1*, and in the three loci previously characterized. These lines were developed by phenotypic and genotypic selection during recurrent backcrossing of four Don-0/L*er* recombinant inbred lines (RILs), as described in Arteaga et al. (2021). Briefly, four Don-0/L*er* RILs (Méndez-Vigo et al. 2016) selected as high FTN were backcrossed to L*er* during three generations, always using a plant with a high phenotypic value per population. F_2_BC_3_ plants (∼40 per population) were thoroughly genotyped with 120 genome-wide SNPs (Méndez-Vigo et al. 2016). Four lines carrying Don-0 alleles in two to four of these loci were crossed to L*er* and *F*_1_ hybrids were selfed. Five *F*_2_ families of ∼200 to 300 plants derived from these crosses were genotyped with markers in these genomic regions (Supplementary Table S3; Arteaga et al. 2021) to obtain lines carrying Don-0 alleles in *MAU1*, *TCL1*, *GL1*, and/or *TRY* in an otherwise L*er* background. The size of Don-0 introgression fragments varied from 4 to 10 mb, final ILs carrying 3% to 18% of their genome from Don-0. The 15 different ILs were named as IL followed by the numbers of the chromosomes containing the introgressed loci with Don-0 alleles (e.g. IL-1, IL-12, IL-123, and IL-1235).

Fine mapping of *MAU1* was carried out using a self-progeny of 1,771 plants derived from an IL heterozygous Don-0/L*er* at *MAU1* and homozygous for Don-0 alleles at *TCL1* and *TRY*. Individual plants of this mapping population were genotyped with 22 markers (Supplementary Table S3) to find recombination events in *MAU1* genomic region. The self-progeny of the recombinants was further genotyped to select homozygous plants for each recombination event. Homozygous recombinant lines were then genotyped for *MAU1* using the average FTN estimated from 10 to 15 sister plants of the self-progeny.

The double mutant line *egl3-1 gl3-1*, in L*er* genetic background, was obtained from NASC (N66490) and has previously been described as carrying EMS loss-of-function mutations that cause premature stop codons in both genes (Zhang et al. 2003). The single loss-of-function mutants *egl3-1* and *gl3-1* were isolated from an *F*_2_ progeny derived from the cross of L*er* with line *egl3-1 gl3-1*, which was genotyped using mutant allele-specific markers (Supplementary Table S3).

### Growth conditions and phenotypic analyses

Plants were grown in pots with soil and vermiculite at 3:1 proportion using growth chambers at 21 °C and a long-day (LD) photoperiod (16 h of cool-white fluorescent light, photon flux of 100 *µ*mol/m^2^s).

For phenotypic analyses of trichome traits, all ILs, or transgenic lines in the same genetic background, were grown simultaneously in the same experiment, including the corresponding control genotypes (mutants, ILs and/or parental accessions). Unless stated otherwise, for all traits, we used four (ILs), or three (transgenic lines) complete blocks designs with randomization, each block containing one pot with six plants per line.

Trichome patterning was measured in leaves, stems, pedicels, and fruits by recording four quantitative traits: leaf trichome density (LTD), stem trichome pattern (STP), pedicel trichome pattern (PTP), and fruit trichome number (FTN). LTD was quantified as the ratio between the number of trichomes in the adaxial side of leaf number 5 to 6, and leaf surface in mm^2^. Leaves were collected and photographed when they were fully expanded, and leaf trichome numbers and surfaces were scored on the photographs using the image analysis software ImageJ (http://imagej.net). STP was measured as the number of internodes between cauline leaves and fruits, developing trichomes. Similarly, PTP was measured as the number of pedicels with trichomes. Only the first 22 internodes or flowers were considered to quantify STP and PTP, respectively. FTN was scored as the number of trichomes in the two valves of the first fully fertile fruit (position 1 to 3 of the main inflorescence), which was counted under a stereomicroscope (30× magnification). Given the low number of trichomes in stems of transgenic lines developed in *egl3 gl3* genetic background, an additional trait, stem trichome number (STN), was quantified in these lines. STN was measured as the total number of trichomes in the whole stem of the main inflorescence.

Trichome branching was quantified in the basal internodes of the main stem, as previously described (Arteaga et al. 2022). Stems were collected and photographed when plants were fruiting to ensure that they were fully elongated. From these pictures, the number of trichomes with one (simple), two, or more than two branches were recorded in the second internode, using the cell counter tool of ImageJ software (http://imagej.net). Trichome branching was then estimated as the proportions of trichomes with different number of branches.

Anthocyanin content in hypocotyls was measured in transgenic lines using a photometric test previously described (Rabino and Mancinelli 1986; Ilk et al. 2015). All lines were measured simultaneously in a single experiment with three replicates per line organized in three complete randomized blocks. Briefly, seeds from each T_3_ transgenic line were sown in three Petri dishes (∼100 seeds per plate) containing a filter paper soaked with demineralized water and incubated 4 d at 4 °C in darkness to break seed dormancy. Thereafter, dishes were transferred to a growth chamber at 21 °C with LD photoperiod. After 5 d, 30 seedlings per plate were incubated during 24 h at 4 °C in 500 *µ*l of acidic methanol (0.5N chloridric acid, 80% methanol). Samples were then diluted with 500 *µ*l of 0.15N chloridric acid, 80% methanol, and incubated 15 min in darkness before measuring the absorbances at 530 and 657 nm. To relativize the amount of anthocyanin to weight, 30 seedlings were weighted from each Petri dish. Anthocyanin content was estimated in units of absorbance per mg of fresh weight, using the formula (A530−(0.25*A657))*1000/weight.

### *EGL3* sequencing, genomic constructs and transgenic lines

An *EGL3* genomic fragment of 6.7 kb was sequenced from Don-0 and L*er* accessions, including: 2.9 kb of promoter and 5′ UTR; 2.9 kb of coding sequence; and 0.9 kb of the 3′ region. To this end, 15 overlapping fragments of 0.8 to 1.2 kb were PCR amplified (Supplementary Table S4) and products were sequenced using an ABI PRISM 3730xl DNA analyser. DNA sequences were aligned using DNASTAR v.17.0 (Lasergene) and alignments were inspected and edited by hand with GENEDOC (Nicholas et al. 1997). Gene sequences are available in GenBank/EMBL databases under the accession numbers PQ367244-PQ367245.

The two genomic fragments sequenced from Don-0 and L*er* were cloned in the pCAMBIA 2300 binary vector (CAMBIA, Canberra, Australia) by standard molecular biology techniques. These fragments were PCR amplified using Phusion high-fidelity DNA polymerase (New England Biolabs, Beverly, USA), cloned in *BamH*I/*Sal*I restriction sites, and checked by sequencing (Supplementary Table S4). Constructs were transferred by electroporation to AGL0 *Agrobacterium tumefaciens* strain (Lazo et al. 1991) and Arabidopsis plants of two genotypes, *egl3 gl3* and IL-25, were transformed by the floral dip method (Clough and Bent 1998). T_1_ transformants were screened by kanamycin resistance and lines carrying single insertions were selected based on resistance segregation in T_2_ families. Ten to 15 independent homozygous T_3_ lines were selected for each construct and genetic background, their transgene and endogenous *EGL3* alleles being verified by PCR before phenotypic analyses (Supplementary Tables S3 and S4).

### *EGL3* phylogenetic and expression analyses

The intraspecific relationships among *EGL3* genomic sequences derived from 235 Iberian genomes previously sequenced (1001 Genomes Consortium 2016; Arteaga et al. 2021) (Supplementary Table S2) were determined with 312 SNPs and indels located within the 6.9 kb spanning between *EGL3* and flanking genes. A neighbor-joining tree was constructed from these polymorphisms using MEGA v.7 (Tamura et al. 2011) and applying 10,000 bootstrap permutations for statistical significances. Structural variants in these genes were genotyped in the 235 Iberian accessions by reanalyzing the alignments in the nucleotide positions of Don-0/L*er* polymorphisms and genotyping with specific indel markers. Allele frequencies of *EGL3* polymorphisms were also estimated from 1,135 worldwide genomes (1001 Genomes Consortium 2016) and 80 African genomes (Durvasula et al. 2017) currently available.

*EGL3* sequences from 23 Brassicaceae plants were obtained from Phytozome v. 13 (https://phytozome-next.jgi.doe.gov) or from *Cardamine hirsuta* genome project (http://chi.mpipz.mpg.de/). The alignment of proteins was obtained with CLUSTAL (https://www.ebi.ac.uk/Tools/msa/clustalo/) and edited by hand with GENEDOC (Nicholas et al. 1997).

For expression analyses, plants were grown as described for phenotypic analyses, but pots contained ∼50 (for leaf samples) or 9 (for stems and flowers) plants. After sowing, pots were placed at 4 °C and short day photoperiod (8 h of cool-white fluorescent light) for seed stratification during 4 d. Thereafter, pots were transferred to a growth chamber with LD photoperiod and 21 °C. Genotypes to be compared (parental accessions, ILs, or transgenic lines in the same genetic background) were grown simultaneously in a single experiment, including three pots per genotype (or nine pots for parents and controls), organized in three randomized blocks. For leaf samples, 14- to 18-d-old rosettes were harvested, whereas for flower samples, buds of the main inflorescences were collected 5 d after flowering initiation (∼ 30- to 35-d-old plants depending on the genetic background). For stem samples, the shoot of the main inflorescence was collected from the same plants used for flowers, after removing cauline leaves, lateral branches, and flower buds. Tissue from the three blocks of each genotype was mixed before RNA isolation using TRIzol reagent according to manufacturer's protocol (Invitrogen). Potential DNA contamination was removed by DNAse digestion and subsequent RNA purification was carried out with high pure RNA isolation kit (Roche). cDNA was synthesized from 3 *µ*g of total RNA using AMV reverse transcriptase (Invitrogen) and dT15 oligonucleotides. *EGL3* expression was analyzed by quantitative real-time PCR (qPCR) (Supplementary Table S4). To avoid amplification differences caused by DNA polymorphisms, primers were designed in gene regions carrying no polymorphism between L*er* and Don-0. The gene encoding ubiquitin-conjugating enzyme 21 (*UBC, At5g25760*) was used as endogenous control for RNA sample standardization (Supplementary Table S4). Genes were amplified with Power SYBR green mix in a 7300 real-time PCR system (Applied Biosystem) and quantified using the standard curve method. Mean and standard errors were derived from three biological replicates (RNA isolated from plants grown in different pots), each of them with three technical replicates (qPCR wells from the same cDNA sample) for parental accessions and ILs; or from four technical replicates for transgenic lines.

### Statistical analyses

Phenotypic and gene expression differences between ILs were tested by mixed general linear models (GLMs), including genotypes as fixed effect factors and lines of the same genotype (nested within genotypes) as random effect factor. In ILs, the additive and interaction effects of *MAU1*, *TCL1*, *GL1*, and *TRY* were tested by GLMs, including the four loci as fixed effect factors. Expression differences between parental accessions and organs were tested by GLMs, including genotypes and organs as fixed effect factors. Differences between transgenic lines were also tested by mixed GLMs, including transgenes as fixed factor, and lines (nested within transgenes) as random factor. The percentage of variance explained by each factor was estimated by the restricted maximum likelihood (REML) method of variance component analysis. These analyses were carried out with the statistical packages SPSS v.27 or Statistica v.8. All statistical analyses are detailed in Supplementary Table S1.

### Accession numbers

Sequence data from this article can be found in the GenBank/EMBL data libraries under accession numbers PQ367244-PQ367245 and in Supplementary Fig. S4.

## Supplementary Material

kiae673_Supplementary_Data

## Data Availability

The data underlying this article are available in the article and in its online Supplementary material.

## Supplementary data

The following materials are available in the online version of this article.

**Supplementary Figure S1.** Trichome pattern of parental and ILs.

**Supplementary Figure S2.** Effect of *egl3* and *gl3* mutations on trichome pattern, trichome branching, and anthocyanin content.

**Supplementary Figure S3.** Leaf trichome density of transgenic lines for *EGL3*.

**Supplementary Figure S4.** Nucleotidic and geographic diversity of *EGL3*.

**Supplementary Table S1.** Summary of statistical analyses in parental, introgression, and transgenic lines.

**Supplementary Table S2.** Geographic, phenotypic, and genotypic information of the Arabidopsis Iberian accessions.

**Supplementary Table S3.** Molecular genetic markers used for the development of ILs and fine mapping of *MAU1*.

**Supplementary Table S4.** Oligonucleotides used for *EGL3* sequencing, cloning, genotyping, and expression analyses.

## Dive Curated Terms

The following phenotypic, genotypic, and functional terms are of significance to the work described in this paper:

EGL3 Gramene: AT1G63650
EGL3 Araport: AT1G63650

AT1G63650 Gramene: AT1G63650
AT1G63650 Araport: AT1G63650

GL2 Gramene: AT1G79840
GL2 Araport: AT1G79840

GL3 Gramene: AT5G41315
GL3 Araport: AT5G41315

trichome Gramene: NCBI
trichome Araport: NCBI

TRY Gramene: AT5G53200
TRY Araport: AT5G53200

MAU1 (MALAMBRUNO 1) Gramene: AT1G63650
MAU1 (MALAMBRUNO 1) Araport: AT1G63650

TCL1 Gramene: AT2G30432
TCL1 Araport: AT2G30432

GL1 Gramene: AT3G27920
GL1 Araport: AT3G27920
