# Supplementary material for: The bHLH transcription factor gene EGL3 accounts for the natural diversity in Arabidopsis fruit trichome pattern and morphology
Source: Plant Physiol. 2024 Dec 22;197(1):kiae673. doi: 10.1093/plphys/kiae673 (PMC11773808; doi:10.1093/plphys/kiae673)
Supplement: kiae673_Supplementary_Data [file kiae673_supplementary_data.zip › MendezVigo_et_al_Supplementary_Data.pdf]

SUPPLEMENTARY DATA

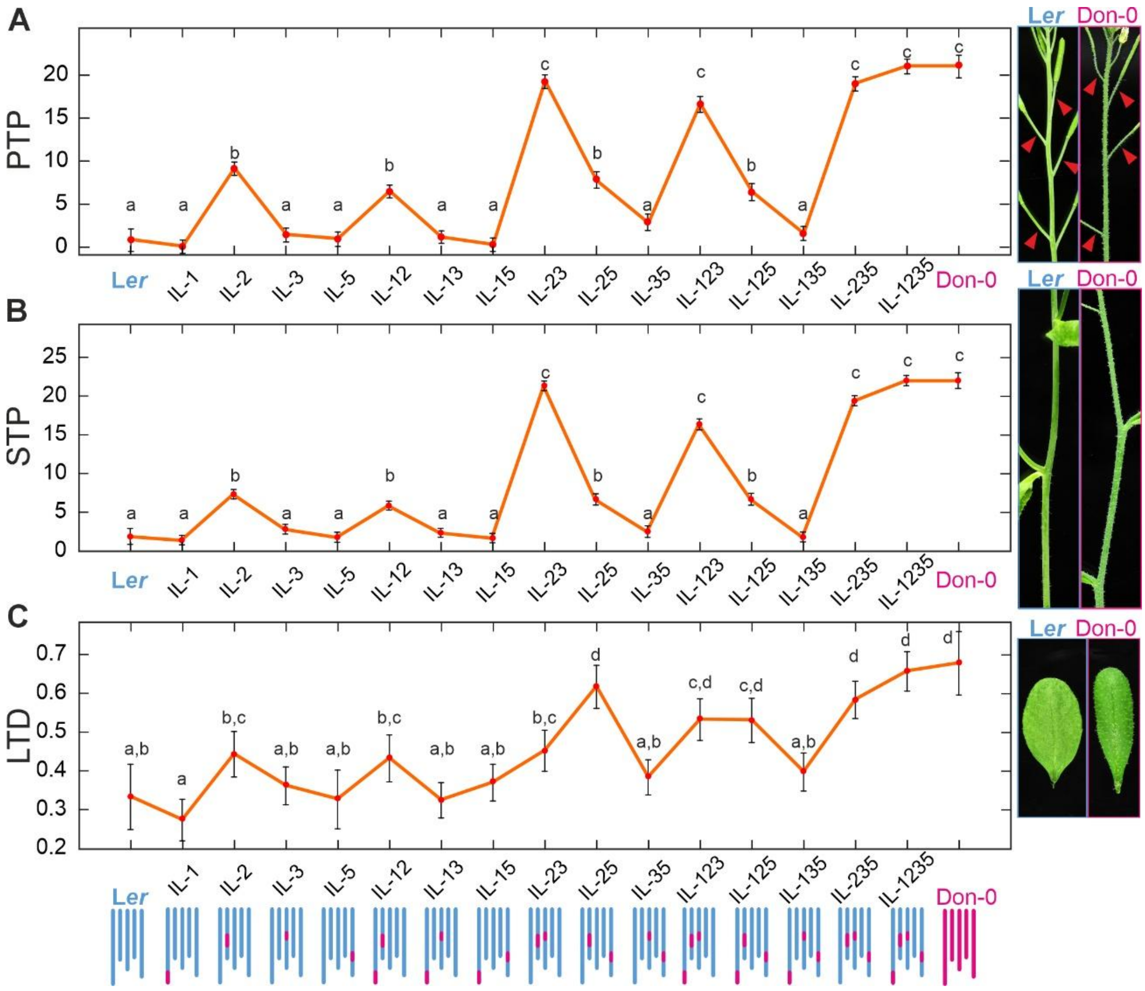

Supplementary Figure S1. Trichome pattern of parental and introgression lines. A) Pedicel trichome pattern (PTP), B) stem trichome pattern (STP) and C) leaf trichome density (LTD) of introgression lines differing in Don-0/L*er* alleles at *MAU1*, *TCL1*, *GL1* and *TRY* genomic regions. PTP and STP were measured as the number of hairy internodes or pedicels in the first 22 internodes of the main inflorescence. Dots and bars correspond to means ± 0.95 confidence intervals of two or three lines per genotype (8-24 plants per line). Representative organs of parental accessions, Don-0 and L*er*, are shown in the right side next to each panel, whereas graphical genotypes of ILs are depicted in the lower part of the figure. Arrowheads in pictures of panel A point to pedicels. In each panel, phenotypic differences among genotypes were tested by mixed linear models, and the same or different letters indicate non-significant and significant differences, respectively, as estimated by Tukey's test (P<0.05).

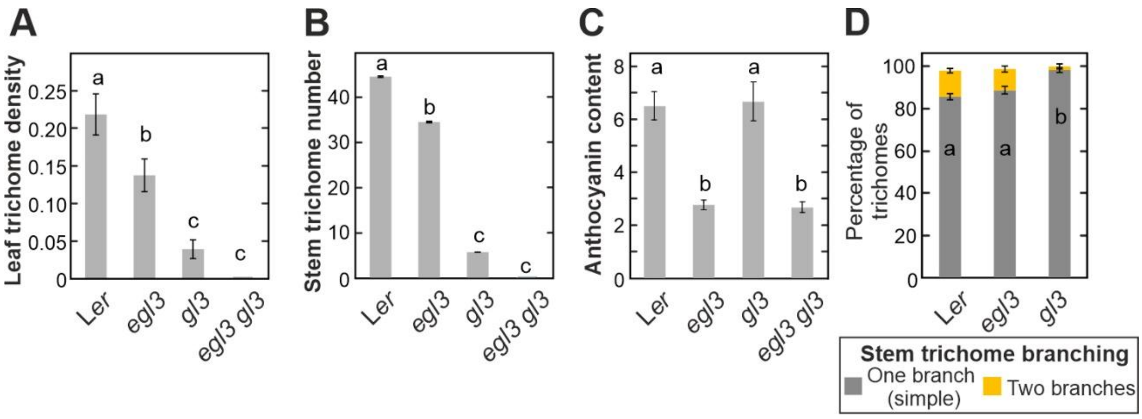

Supplementary Figure S2. Effect of *egl3* and *gl3* mutations on trichome pattern, trichome branching and anthocyanin content. Leaf trichome density (A) and stem trichome pattern (B) of L*er* control and *egl3*, *gl3*, *egl3 gl3* mutants. Bars depict the mean ± SE of 8-15 plants per genotype. C) Hypocotyl anthocyanin content measured as units of spectrophotometer absorbance per mg of fresh weight, in L*er* control and *egl3*, *gl3*, *egl3 gl3* mutants. D) Stem trichome branching of L*er* control, as well as of *egl3* and *gl3* mutants. In this panel, the double mutant *egl3 gl3* was not included because it is glabrous (see A, B). In A-C, each bar represents the mean percentage ± SE of trichomes with one or two branches in 15 plants per genotype. In D, the bar of each genotype shows the percentage ± SE of trichomes of each branching class quantified in 15 plants per line. In all panels, phenotypic differences among genotypes were tested by general linear models, and the same or different letters indicate non-significant and significant differences, respectively, as estimated by Tukey's test (P<0.05).

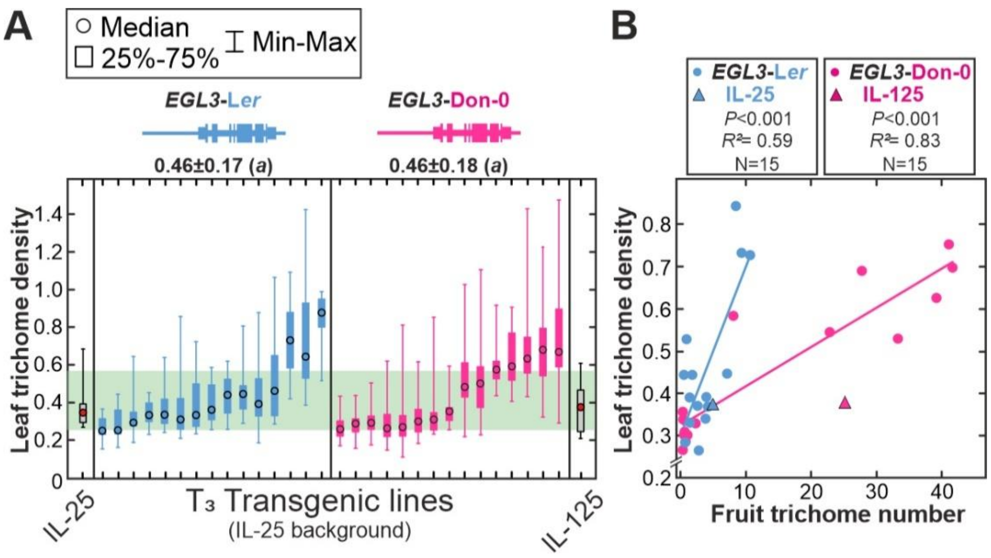

Supplementary Figure 3. Leaf trichome density of transgenic lines for *EGL3*. A) Leaf trichome density of independent homozygous transgenic lines carrying L*er* or Don-0 genomic constructs of *EGL3*, in IL-25 genetic background. Each bar represents the phenotypic distribution of 10-18 plants from an independent homozygous transgenic line. B) Relationship between leaf and fruit trichome patterns in transgenic lines carrying L*er* or Don-0 genomic constructs of *EGL3*, in IL-25 genetic background. Leaf trichome density is the number of trichomes per leaf mm^2, whereas fruit trichome number is the number of trichomes in the first fruit. In A, drawings of *EGL3* genomic constructs are shown on top of the panel, with magenta (Don-0) and blue (L*er*) colors depicting different coding and regulatory regions in both transgenes. In these panel, transgenic lines are arranged from low to high mean phenotypic values, and 95% confidence intervals for untransformed controls are shown as green-shaded areas. In A, phenotypic differences among genotypes were statistically tested by mixed linear models; the same or different letters on top of each panel indicate non-significant or significant differences, as tested by Tukey's test (P<0.05). In B, linear regression analyses were used to test the relationships between leaf and fruit trichome traits.

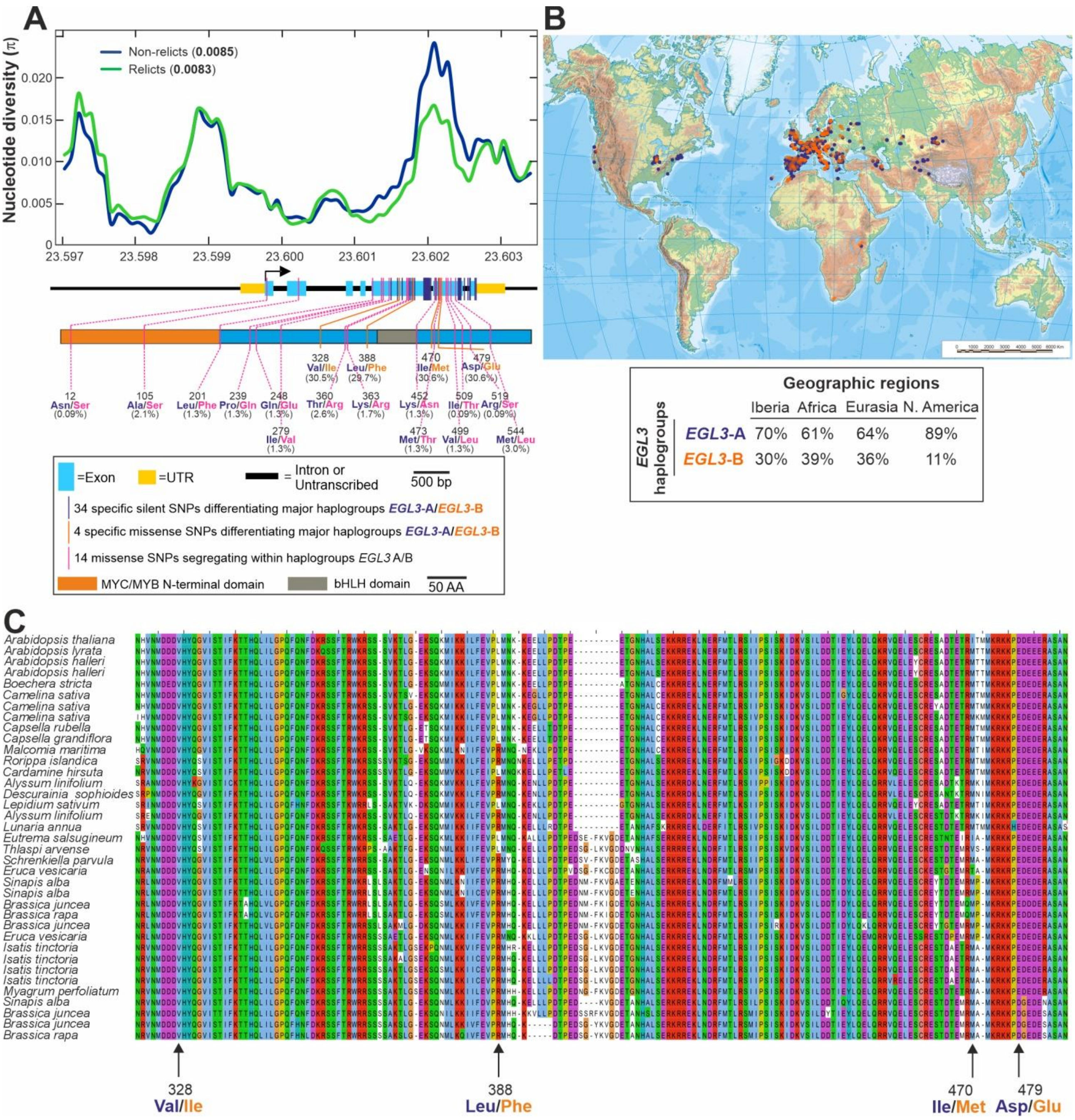

Supplementary Figure 4. Nucleotidic and geographic diversity of *EGL3*. A) Sliding window plot of the nucleotide diversity in 235 Relict and Non-relict accessions from the Iberian Peninsula. The average nucleotide diversity (π) of each group of accessions is shown within the graph. *EGL3* genomic and protein structure is shown below the abscissa axe, including the location and the total number of SNPs differentiating the two major haplogroups *EGL3*-A and *EGL3*-B. Missense mutations differentiating haplogroups *EGL3*-A and *EGL3*-B, or segregating within these haplogroups, are indicated with different colors below EGL3 protein structure. B) World-wide geographic

distribution of the two major *EGL3* haplogroups. Frequency of haplogroups in different world regions is shown in the legend. C) Sequence alignment of 37 EGL3 proteins from Brassicaceae. The sequences of EGL3 carboxi-half from 23 Brassicaceae species belonging to 13 different tribes are shown. The four amino acid substitutions differentiating the two major *EGL3* haplogroups are indicated with arrows. Genbank or Phytozome accession numbers of the protein sequences included are as follow: *Arabidopsis thaliana* (AT1G63650.1/AF013465.1), *A. lyrata* (AL2G11580.t1), *A. halleri* (Ah2G01730.1 and Ah2G01590.1), *Camelina sativa* (CsAmes1043.07G372000.1.p, CsAmes1043.09G400400.1.p and CsAmes1043.16G360300.1.p), *Capsella rubella* (Carub.0002s0120.1.p) and *C. grandiflora* (Cagra.2104s0007.1.p) from Camelineae tribe; *Boechera stricta* (Bostr.29223s0199.1.p) from Boechereae; *Malcolmia maritima* (Mamar.0007s0192.1.p) from Physarieae; *Cardamine hirsuta* (CARHR047690) and *Roripa islandica* (Roisl.0010s0189.1.p) from Cardamineae; *Alyssum linifolium* (Alyli.0063s0184.1.p and Alyli.0035s0171.1.p) from Alysseae; *Descurainia sophioides* (Desop.0005s0134.1.p) from Descuraineae; *Lepidium sativum* (Lesat.0071s0288.1.p) from Lepidiaeae; *Lunaria annua* (Luann.0159s00083.1.p) from Biscutelleae; *Eutrema salsugineum* (Thhalv10023356m) from Eutremeae; *Thlaspi arvense* (Thlar.0003s0129.1.p) from Thlaspideae; *Schrenkielleae parvula* (Sp2g01010.1) from Schrenkielleae; *Eruca vesicaria* (Eruve.1157s0020.1.p and Eruve.0507s0023.1.p), *Sinapis alba* (Sialb.3217s0007.1.p, Sialb.1166s0033.1.p and Sialb.0010s0642.1.p), *Brassica juncea* (Braju.12G189400.1.p, Braju.12G204500.1.p, Braju.09G142000.1.p and Braju.09G127300.1.p), and *Brassica rapa* (Brara.I01388.1.p and Brara.I01246.1.p) from Brassiceae; and *Isatis tinctoria* (Isati.0466s0029.1.p, Isati.0498s0023.1.p, Isati.10854s0002.1.p and Isati.2003s0015.1.p) and *Myagrum perfoliatum* (Myper.0014s0801.1.p) from Isatideae tribe.
