# Supplementary material for: The bHLH transcription factor gene EGL3 accounts for the natural diversity in Arabidopsis fruit trichome pattern and morphology
Source: Plant Physiol. 2024 Dec 22;197(1):kiae673. doi: 10.1093/plphys/kiae673 (PMC11773808; doi:10.1093/plphys/kiae673)
Supplement: kiae673_Supplementary_Data [file pp.02635.2024-s06.pdf › pp.02635.2024-s06.pdf]

Plant Physiology®

# The bHLH transcription factor gene EGL3 explains Arabidopsis natural diversity in fruit trichome pattern and morphology

Belen Mendez-Vigo, Noelia Arteaga, Alba Murillo-Sánchez, Sonia Alba, and Carlos Alonso-Blanco

**Review Timeline:**

| | |
|---|---|
| Submission Date: | 08-Oct-2024 |
| Editorial Decision: | 22-Nov-2024 |
| Revision Received: | 02-Dec-2024 |
| Accepted: | 03-Dec-2024 |

22-Nov-2024

Dr. Carlos Alonso-Blanco
Consejo Superior de Investigaciones Cientificas
Plant Molecular Genetics
Madrid, Sp N/A
Spain

RE: The bHLH transcription factor gene EGL3 explains Arabidopsis natural diversity in fruit trichome pattern and morphology

Dear Dr. Alonso-Blanco:

We have received reviews of your manuscript, which you may view at the end of this letter. On the basis of the advice received and my own evaluation, I believe that, pending revision, this article may ultimately be acceptable for publication in Plant Physiology.

In particular, please pay attention to the below two questions:
1) The section where you explore the impacts of multiple EGL3 gain-of-function could be clearer. It is unclear whether you have separately analysed the impacts of the cis-regulatory mutations vs structural mutations and their effects, though one gets a general understanding that it is structural polymorphisms that are contributing to the phenotypic differences.
2) Fig 2. The colour scheme in Fig 2 gives an impression of the promoter region being the same (EGL3 in black) but the reading of the text suggests that the promoter region is from respective accessions. Perhaps the same comment goes for some of the other figures too. Please clarify.

When you are ready to submit your revision, click the below link and find the red arrow under your Author Tasks heading for this revision submission.

If you want to visit this version of the manuscript, you may click the link below and then click on the folder that reads "Post Decision Manuscripts".

Link Not Available

When uploading the revised version of this article, please be sure to include as complete an answer as possible to the referees' criticisms during the first round of reviews in the "response to reviewers" section.

Plant Physiology considers a revision for up to 60 days after a manuscript has been provisionally accepted; limited extension of this timeframe is at the discretion of the Monitoring Editor. A reminder notice will be sent to you 2 weeks before the deadline, at which time you may choose to contact the editorial office to request an extension; if so, please provide a justification for your request. After the expiration of this deadline, the article would need to be submitted as a resubmission and would be handled as such.

Thank you very much for giving us an opportunity to review this work. I look forward to receiving the next version.

Sincerely,

Dr. Ya-Long Guo
Monitoring Editor, Plant Physiology

----

Want to add this revision deadline to your calendar? Click below!

------------------------------------------------------------------------- Reviewer comments:
Reviewer #1 (Comments for the Author):

The manuscript by Mendez-Vigo et al describes an excellent study on the genes/alleles hat control the formation of trichomes

on fruits. This in Arabidopsis novel trait was identified for the first time in a previous publication by the group of Alonso-Blanco. In the present paper they identified the identity and described the allelic variation underlying of one of the essential loci that is responsible for this trait. Although the QTL as such was described before the nature of the allelic variation at the molecular level and the details of its interaction with alleles of three other genes had not been described before. The complexity of the study was the genetic interaction among this specific MAU3 locus (which appeared to be EGL3) with specific alleles at three other loci. This required firstly a detailed genetic analysis which was done with large number of so-called introgression lines and the use of specific genetic backgrounds for the introduction of specific alleles by transformation followed by detailed phenotypic description and gene expression analysis of a large number of transgenic lines. The discussion speculates on the role of variation in trichome numbers and also on the world wide allelic variation at the locus in relation to its origin and the phenotype.
This reviewer has no suggestions for improvements of the manuscript except a few more editorial comments.
Specific comments.
Line 145 172. Are the trichomes not located on instead of in the siliques. In suggests within and does not tell about the surface nature of the phenotype. Maybe more correct in epidermal tissues .
Line 381-407. It was less clear to this reviewer that (not) all fruit trichome EGL3 alleles have the Asn/Ser12 substitution. If so this is an additional proof that this is the causal mutation,
Line 529. Why not mention what is specific for Il 25?

Reviewer #2 (Comments for the Author):

This study explores a locus MALAMBRUNO 1 (MAU1), a locus affecting the trichomes in Arabidopsis fruits and its genetic interactions with other genes, such as GL1. Authors show that MAU1 encodes transcription factor EGL3 and natural variation at EGL3 is responsible for this variation in the Don-0 accession of Arabidopsis.

Previously Alonso-Blanco group uncovered the MAL loci that contribute to fruit trichomes and here they clone and characterise one of these loci (MAL1). Nice genetic analysis allowed them to identify the requirement of other genes (TCL1/TRY/GL1) and then by selecting for lines that are segregating at MAL and homozygous for TCL1/TRY, they were able to positionally clone MAL1 as EGL3. Using transgenics, they are able to show a quantitative difference in complementation, with the Don-0 allele conferring stronger phenotypic complementation.

The paper is clean till they show that there is no difference in gene expression that accounts for the allelic variation. However, the other parts of the specific polymorphisms are more inferential rather than experimental. Having said that, it still is consistent with the genetic data and the haplotypic effects are clearly shown.

The section where the authors are exploring the impacts of multiple EGL3 gain-of-function could be clearer. It is unclear whether the authors have separately analysed the impacts of the cis-regulatory mutations vs structural mutations and their effects, though one gets a general understanding that it is structural polymorphisms that are contributing to the phenotypic differences.

Fig 2. The colour scheme in Fig 2 gives an impression of the promoter region being the same (EGL3 in black) but the reading of the text suggests that the promoter region is from respective accessions. Perhaps the same comment goes for some of the other figures too. Please clarify.

In the following point by point revision we describe the precise changes that we have made in the new version of the manuscript. All changes are highlighted by showing them in yellow color in the file corresponding to the Main text, as well as in the Supplementary Figures and in Figure 2 files.

**Reviewer 1**

General comment

"*This reviewer has no suggestions for improvements of the manuscript except a few more editorial comments."*

We thank the positive view and kind words of Reviewer 1 on our manuscript.

Specific comments

1. "*Line 145 172. Are the trichomes not located on instead of in the siliques. In suggests within and does not tell about the surface nature of the phenotype. Maybe more correct in epidermal tissues.*"

To avoid misunderstanding, we follow the Reviewer suggestion, and we have now changed the preposition "in" with "on" (page 5, line 145), and we have added "in the epidermis of " (page 5, line 158).

2. "*Line 381-407. It was less clear to this reviewer that (not) all fruit trichome EGL3 alleles have the Asn/Ser12 substitution. If so this is an additional proof that this is the causal mutation?"*

This point of Reviewer 1 is related with the main comment of Reviewer 2 (see below). We have now thoroughly rewritten this section and we have clearly stated that only two accessions have Ser12 mutation (page 12, lines 374-377). In addition, we now indicate more precisely how *EGL3* alleles are classified in this section by adding that "accessions were genotyped as H for *EGL3* when they belong to potential *EGL3* gain-of-function subhaplogroups A1, A2 and A3, independently of the polymorphisms differentiating them."

3. *"Line 529. Why not mention what is specific for Il 25?"*

Following the Reviewer's suggestion we have now indicated the presence of functional *GL3* alleles of this background by adding "IL-25 genetic background with an active *GL3* allele" (page 16, line 537).

**Reviewer 2**

General comment

*“The paper is clean till they show that there is no difference in gene expression that accounts for the allelic variation. However, the other parts of the specific polymorphisms are more inferential rather than experimental. Having said that, it still is consistent with the genetic data and the haplotypic effects are clearly shown.”*

We thank the positive comments of Reviewer 2. We agree that the last section of the Results section is more inferential and speculative, but as indicated by Reviewer, we still believe that is consistent and informative on the diversity of *EGL3*.

Specific comments

1*.”The section where the authors are exploring the impacts of multiple EGL3 gain-of-function could be clearer. It is unclear whether the authors have separately analysed the impacts of the cis-regulatory mutations vs structural mutations and their effects, though one gets a general understanding that it is structural polymorphisms that are contributing to the phenotypic differences.”*

Following the Reviewer suggestion we have thoroughly rewritten the section describing the diversity in potential structural and regulatory polymorphisms of *EGL3* (page 12, lines 370-383). Similarly, as mention in Reviewer 1 comment, we have now clarified how accessions are classified for *EGL3* gain-of-functions to estimate their effect on the fruit hairy phenotype (page 12, lines 396-400).

2.” *Fig 2. The colour scheme in Fig 2 gives an impression of the promoter region being the same (EGL3 in black) but the reading of the text suggests that the promoter region is from respective accessions. Perhaps the same comment goes for some of the other figures too. Please clarify.*”

As the Reviewer indicates, and stated in the main text (page 7, lines 224-226; page 9, lines 300-301), our genomic constructs include the promoter and regulatory regions from each accession. We think that the Reviewer has misinterpreted the name of our alleles (*EGL3*-Don-0 and *EGL3*-L*er*) as names of constructs, interpreting the black gene name as promoter region, and the alleles as coding. This interpretation seems very strange because it is used throughout the text of the manuscript. However, to avoid such misinterpretation, we have now added a description of the genomic constructs in the legend of Figure 2 stating “drawings of *EGL3* genomic constructs are shown on top of each panel, with magenta (Don-0) and blue (L*er*) colors depicting different coding and regulatory regions in both transgenes”. Similarly, we have added a similar sentence in the legend of Supplementary Figure 3.

03-Dec-2024
Dr. Carlos Alonso-Blanco
Consejo Superior de Investigaciones Cientificas
Plant Molecular Genetics
Madrid, Sp N/A
Spain

MSID: PP2024-RA-02635R1
MS TITLE: The bHLH transcription factor gene EGL3 explains Arabidopsis natural diversity in fruit trichome pattern and morphology

Dear Dr. Carlos Alonso-Blanco:

I am pleased to inform you that your revised manuscript "The bHLH transcription factor gene EGL3 explains Arabidopsis natural diversity in fruit trichome pattern and morphology" has been accepted for publication in Plant Physiology. Your manuscript will be checked for consistency with journal formatting and image standards by one of our Science Editors, who will contact you shortly. They may have a number of queries and suggestions for you. Please keep in mind that they work to increase the online visibility of your article and its attraction for readers from the widest of backgrounds. You will have the opportunity to review any revisions, and I hope you will work constructively with them to get the most out of your research. You will also have the opportunity to review all changes when you receive the author proofs for your article.

Once validated for publication, your article will be sent to a Science Editor for a final review of your manuscript and source files. After your final files are uploaded, your article will appear in the next available issue and the online Preview version of your article will be posted after your signed Copyright and Checklist forms and final source files are received.

Please be sure to include the following in your manuscript file:
Harvard style citations
List of all author contributions and funding information at the beginning of manuscript after the titles and author list.
List all supplemental materials associated with the article, with titles, at the end of the ms, before the references and acknowledgments section.
Complete Figure Legends for all figures appearing in the article listed before the Literature Cited.

**LICENSE INFORMATION** Shortly after your paper is sent to production, you will receive an email from our publisher's author support team, **SciPris**, with information on article processing charges, as well as the types of Open Access license you would be able to purchase. The accepted version of your manuscript will not appear on our Advance Articles page until the license is selected and signed, so please look out for an email from SciPris. For more information see the Article Publication Fees section of our author guidelines: https://academic.oup.com/plcell/pages/General-Instructions

Finally, we encourage your submission of artwork for the journal cover. If there is an image or illustration related to your paper that you would like to have considered, please email it in .pdf or .tif format to clowe@aspb.org. An image title, brief, 2-3 sentence description of the image, and the name of the person credited for the image are also encouraged. If your image is selected to appear on the online cover of the journal, you will be contacted with the scheduled issue date and further information.

Thank you and congratulations on your Plant Physiology paper!

Sincerely,
Dr. Ya-Long Guo
Monitoring Editor, Plant Physiology

=====
FIRST AUTHOR PROFILES
=====

We recognize the hard work that goes into being First Author of a Plant Physiology paper by publishing a "First Author Feature" for each First Author alongside your paper.

If the first author(s) would like to submit information for a feature on Facebook and Twitter, fill out the form by clicking on the link

below:

Plant Physiology First Author Feature Form: https://forms.office.com/r/ZBCGZfhm8F
If you cannot access the form through the link above, please contact Rachel at rbelsky@aspb.org.

To upload an image of yourself to accompany your profile, please put your photo in the correct journal folder at the link below. You must label your image: Author First Name Last Name Article Title.

First Author Profile Image Dropbox: https://www.dropbox.com/scl/fo/xz029p7qjr2jml2rp2kmq/h?dl=0&rlkey=6fkxcaqmxx35ap152b2osy1fz

=====
IMPORTANT REMINDER: PEER REVIEW REPORTS
=====

If you opted to publish a peer review report along with your article during the original submission process, it will be prepared by the editorial staff and publicly posted with your manuscript, inside the zip file that contains any other supplemental material. As a reminder, the peer review report is a public record of all comments from editors and reviewers, as well as your prior responses, as you received them in the decision letters for each draft of your manuscript. If you agreed to publish this report and have changed your mind, or are not sure if you selected this option, please contact the editorial office as soon as possible before signing the license agreement from our publisher.

---------------------------------------------------------------------------- Reviewer comments:
Reviewer #1 (Comments for the Author):

No further questions. All reviewer comments are dealt with

Reviewer #2 (Comments for the Author):

Authors have addressed all comments. I would still perhaps not differentiate EGL3 gene name in black and then Ler/Don in varied colours, perhaps, I would use the same colours to present the gene name as well. But it is the authors paper and I am happy for them to present the way they think it would reach more people. Nice work!
